# Genomic screening of eight antibiotic-resistant Pseudomonas isolated from rainbow trout (Oncorhynchus mykiss)

**DOI:** 10.1099/acmi.0.001029.v3

**Published:** 2026-02-11

**Authors:** Kenny Oberlé, Gaëlle Bednarek, Claude Rispe, Ségolène Calvez

**Affiliations:** 1 Oniris, INRAE, BIOEPAR, 44300 Nantes, France

**Keywords:** antibiotic resistance, mobile genetic elements, *Pseudomonas*, whole genome

## Abstract

Open aquaculture systems represent a strategic environment for the study of antibiotic resistance dynamics, given the interactions between bacteria from humans, animals and the environment. The genomic investigation of eight *Pseudomonas* isolated from rainbow trout in a previous study demonstrated that non-wild-type and resistance phenotypes to several antibiotics (oxytetracycline, sulphonamides and florfenicol) were predominantly attributable to the presence of related genes [*tet(Y)*, *sul2* and *floR*]. Phylogenetic analyses revealed that several species of *Pseudomonas iridis* harboured these genes on mobile genetic elements, including integrative conjugative elements (ICE) on the chromosome or plasmids with a high degree of sequence similarity (>99%) between the genetic structures. Furthermore, comparisons between isolates with low and high MIC values to colistin showed mutations in the amino acid sequences of the PhoP/Q two-component system and a lack of the pmrA/B system. A wide diversity of known LPS-modifying genes involved in colistin resistance was also detected in resistant isolates. This study provided insights into the dynamics of antibiotic resistance in aquaculture systems. It demonstrated the presence of genes located on an ICE inserted into a chromosome or plasmid in *P. iridis*, which was isolated from healthy rainbow trout in different farms within the same watershed. Our study raises questions about the ability of environmental *Pseudomonas* bacteria to spread their antibiotic resistance genes to other bacterial species that are of interest in terms of human or animal surveillance.

**Impact Statement**

Despite the restricted number of bacterial isolates under investigation, we were able to identify a genetic phenomenon occurring within a bacterial species. These findings encourage further inquiry into the prevalence of such occurrences in aquaculture systems, with the objective of enhancing the One Health surveillance of antibiotic resistance.

## Data Summary

Raw reads and assembled genomes are available in BioProject PRJNA1136410. Genome accession numbers available in the NCBI website are CP162581, CP162582, CP162583–CP162584, CP162585–CP162588, CP162589, CP162590–CP162591, CP162592 and CP162593–CP162597. All scripts used in this study are available at https://doi.org/10.5281/zenodo.17404976.

## Introduction

The emergence of antibiotic-resistant pathogens is a growing global concern with the potential to cause 10 million deaths per year by 2050 [1]. This global health issue is increasingly being studied using the One Health approach, with the objective of gaining a deeper understanding of the emergence and spread of resistance between humans, animals, the food chain and the environment [2,3]. To illustrate, the Tricycle protocol, as proposed by the World Health Organization, is focused on extended spectrum beta-lactamase-producing *Escherichia coli* to facilitate surveillance on a global scale. Nevertheless, a comparable initiative to monitor the prevalence of other bacterial indicators would be a valuable addition to the existing research. The genus *Pseudomonas* is ubiquitous, occurring in many environments, particularly soil [4,6] and water [7,9]. However, it is also a significant opportunistic pathogen causing infections in humans, animals and plants like species *Pseudomonas aeruginosa*, *Pseudomonas fluorescens*, *Pseudomonas tructae* and *Pseudomonas syringae* [10,13]. The diverse ecological habitats of *Pseudomonas* populations facilitate interactions with numerous other bacterial species, resulting in the exchange of genetic material.

Open aquaculture systems are based on fish farming in natural water bodies (oceans, estuaries, rivers, etc.) where food-producing fish are exposed to contaminants (chemical or microbiological) from human activities or terrestrial animal production brought by water. A number of studies have indicated that antibiotic-resistant *Pseudomonas* are frequently found in association with fish in such environments [14,16]. Furthermore, the *in vitro* transfer of plasmid-harbouring antibiotic resistance genes from *Pseudomonas* isolated from Chilean salmonid farms has been demonstrated, thereby providing evidence of the putative dynamic of genetic exchanges in aquaculture environments [17]. In our previous research, we demonstrated that a subset (8 out of 51) of *Pseudomonas* isolated from rainbow trout exhibited high MICs across one (polypeptides) to four different antibiotic classes (tetracyclines, phenicols, sulphonamides/trimethoprim and cephalosporins) [16]. The resistance phenotypes observed in response to these compounds can be attributed to the influence of multiple gene families. Regarding colistin (polypeptide class), the mechanisms of resistance are allowed by cell wall biosynthesis coding genes, which are responsible for phosphoethanolamine (*eptA*, *eptB*, *eptC* or *cptA*) and/or 4-amino-4-deoxy-l-arabinose (*arnBCADTEF* operon) addition to LPS. In Gram-negative bacteria, it has been established that activation of these genes was under the control of two-component systems named PhoP/PhoQ and PmrA/PmrB [18]. The RstA/RstB two-component system has also been identified as a regulator of the dgkA-eptA operon and the colistin-resistant phenotype in *Vibrio* isolates [19]. In addition, plasmid-associated genes, designated *mcr*, are capable of adding phosphoethanolamine [20]. With regard to tetracyclines, a considerable number of genes (*tet*) have been identified in bacteria [21]. These can persist in aquaculture farms without the application of selection pressure [22]. Additionally, multiple genes have been identified as contributing to phenicol resistance. However, *floR* appears to be the most prevalent in aquaculture settings, particularly in the context of florfenicol usage [23,24]. The presence of *sul* or *dfr* genes is often cited as the explanation for sulphonamide and trimethoprim resistances, respectively. However, some resistance phenotypes have been attributed to mutations in dihydrofolate reductase structural genes (namely, *fol*) [25,26]. The majority of these genes or phenotypes, particularly those associated with tetracyclines and sulphonamides, have been observed to be linked to mobile genetic elements, particularly plasmids within fish farms [17,27]. Considering the potential for genetic transfer within the *Pseudomonas* genus and the risk of transmission to opportunistic pathogens affecting animals and humans, it was essential to ascertain which genes were responsible for these phenotypes and whether mobile genetic elements, such as integrative conjugative elements (ICEs) or plasmids, were involved. This would facilitate a deeper understanding of the dynamics of exchange in aquaculture systems. The objective of this study was to establish two key relationships: first, the relationship between phenotypes and genotypes for resistance to antibiotics and, second, the description of genetic structures harbouring resistance genes where appropriate. To achieve this, eight antibiotic-resistant (high MIC values and/or multiple resistant) out of 51 *Pseudomonas* isolated on fish were selected for genomic analyses.

## Methods

### *Pseudomonas* isolates included in the study

In a previous study, 51 *Pseudomonas* were isolated from rainbow trout fillets and characterized in terms of their MICs to different antibiotics [16]. Among these, eight were selected for this study due to their multiple antibiotic resistance and/or high MICs. Individual MICs of the eight isolates named A0308 (A03RPs3-08), A1230 (A12RPs2-30), A1437 (A14RPs3-37), B16120 (B16FPs1-120), B19125 (B19FPs3-125), B21128 (B21FPs3-128), B22129 (B22FPs1-129) and B26140 (B26FPs3-140) were extracted from the previous study (Table 1). MIC assessment was performed according to the protocol described in our previous work focusing on antibiotic resistance phenotypes [13]. For the sake of clarity, it should be noted that the letters A and B in the isolate IDs indicate the location of fresh dead fish sampled in two different farms (A or B) located along the same river, in accordance with the sampling strategy previously described [28].

**Table 1.** MICs of *Pseudomonas* isolates to five antibiotics

Isolate	MIC value (µg ml^−1^)				
	OTC	FFC	TMP/SXT	CAZ	COL
A0308	256	>1,024	>512/9,728	32	2
A1230	64	>1,024	256/4,864	8	2
A1437	256	>1,024	>512/9,728	16	2
B16120	4	128	1/19	16	16
B19125	2	64	4/76	<4	>1,024
B21128	64	512	128/2,432	8	2
B22129	2	64	2/38	8	512
B26140	64	512	128/2,432	8	2

CAZ, ceftazidime; COL, colistin; FFC, florfenicol ; OTC, oxytetracycline ; TMP/SXT, trimethoprim/sulfamethoxazole.

### DNA extraction procedure and quantification

Prior to DNA extraction, each isolate was stored at −80 °C and subsequently cultivated in tryptic soy broth (Biokar Diagnostics, France) at 25±1 °C for 18 h under agitation (87 rotations per minute). The OD was then assessed at 600 nm using a spectrophotometer (Genesys20, Thermo Fisher Scientific™, USA), after which 100 µl of the broth was plated on tryptic soy agar medium for 24 h at 25 °C.

Two distinct DNA extraction procedures were employed to procure the biological material. Each one was spaced from 1 month. The first one was conducted in accordance with the protocol outlined in the DNeasy Blood and Tissue kit, as recommended by the manufacturer (Qiagen Sciences, USA). The DNA extracted by this protocol was used for Illumina sequencing (short reads). The second one was based on the protocol of the Wizard® Genomic DNA Purification kit, as recommended by the manufacturer (Promega Corporation, USA). The DNA extracted using this protocol was subsequently used for Oxford Nanopore Technology sequencing (long reads).

The quantity of all DNA samples was determined using two distinct methods: the Qubit Fluorometric Quantification (Thermo Fisher Scientific™) with dsDNA high-sensitivity assays and the NanoDrop Spectrophotometer (Thermo Fisher Scientific™). For long reads, the 260/280 and 260/230 ratios were monitored and found to fall within the range of 1.8–2 and 2–2.2, respectively. Further, the integrity of the DNA fragments was evaluated through electrophoresis using the GeneRuler High Range DNA Ladder (Thermo Fisher Scientific™) as a molecular weight marker. The gel electrophoresis was conducted using a 0.4% agarose gel with 1X Tris-Acetate-EDTA at 3 V cm^−1^ for 1.5 h.

### Whole-genome sequencing and analyses

The DNA of each *Pseudomonas* isolate was sequenced using both the Illumina (MiSeq System^®^) and Oxford Nanopore (GridION nanopore sequencer) technologies, in order to obtain paired-end short reads and long reads, respectively. This aspect of the work was performed by the GeT-PlaGe genomic and transcriptomic platform (https://get.genotoul.fr/la-plateforme/get-plage/, Toulouse, France). All subsequent analyses of the raw reads and assembled genomes were conducted using the Genotoul bioinformatic platform Toulouse Occitanie (https://bioinfo.genotoul.fr/).

### Whole-genome assembly

To avoid issues with different read sets (short and long), we decided to use only long reads for genome assembly and then use short reads for polishing and correctness criteria. In all sections below, default settings are used for all bioinformatic analyses unless otherwise stated.

The quality control and preprocessing of each short and long read were conducted using the fastp v0.23.2 [29] and filtlong v0.2.1 [30] software, respectively. The Filtlong tool was employed to eliminate long reads of insufficient length (less than 1 kb) and to exclude the lowest-quality 5% of the dataset. The Trycycler tool (v0.5.4) was used to generate consensus assemblies using filtered long reads [31]. In seven out of the eight isolates, subsampling reads (*n*=12) were generated prior to the assembly step. In the case of the B26140 isolate, a greater number of subsampling reads (*n*=24) were generated due to the inherent complexity of the assembly process. For all isolates, the assemblers Flye (v2.9.2), Raven (v1.8.3), NextDenovo (v2.5.2) and Canu (v1.9) were employed on three maximally independent subreads [32,35]. In the case of the B26140, the previous assemblers were augmented with the addition of Miniasm (v0.3) coupled to Minipolish (v0.1.3) and Necat (v20200803) [36,38]. Following the processing of each assembler on the various subreads, the contigs were clustered using the Trycycler cluster command. The relevant clusters were selected based on two criteria: firstly, their size (~6 Mb for the genome) and, secondly, their weak representation in the different assemblers (for example*,* only 1 or 2 contigs found among the possible 12 or 24 ones). Some contigs with length issues (dependent on the assembler used) were manually trimmed in order to facilitate the reconciliation step of Trycycler (see the supplementary data for further details). Prior to the reconciliation step, dot plots presenting pairwise combinations of sequences were analysed to define if there were any issues with one or more contigs. Furthermore, these dot plots were employed to ascertain the presence of linear DNA molecules, such as putative plasmids. During the reconciliation step, some contigs were removed due to the presence of circularization issues and/or the lowest overall pairwise identities (the default value in Trycycler) or 1 kbp pairwise identities (<85%). A total of 3 to 18 contigs were retained per cluster for final reconciliation (Supplemental data), and a consensus contig was made for each one. A preliminary polishing step was performed on each consensus sequence with Medaka v1.9.1 [39], followed by two additional steps with short reads using Polypolish v0.5.0 [40] and Polca, which is part of the MaSuRCA assembler v4.1.0 [41].

### Quality control of genome assemblies using the 3C criterion

The contiguity, completeness and correctness of the assembly were evaluated in accordance with the protocol adapted from Molina-Mora *et al*. [42]. The quality and contiguity of the assembly were analysed using QUAST v5.2.0 [43] at the contig level. It was anticipated that the targeted values for the total length of the assembly would range from 6.0 to 6.5 Mb, with a single contig and an N50 approaching the genome size. Completeness was checked by utilizing the Benchmarking Universal Single Copy Ortholog (BUSCO) tool v5.7.1 [44] to identify single-copy orthologue genes and assess gene content in accordance with the 782 genes present in the Pseudomonales odb10 database. The circularization of each contig was evaluated using the Trycycler tool (as previously described) and the number of CDS, rRNA, transfer-messenger RNA (tmRNA) and tRNA was determined through the automatic annotation of complete genomes using Prokka v1.14.5 [45] and Bakta v1.8.2 [46]. To ensure correctness, short reads were mapped to polished genome assemblies using BWA-MEM2 v2.2.1 [47]. Samtools v1.20 [48] was employed to convert the mapped files, after which Qualimap v31-08-20 [49] was applied to evaluate the general error rate (target value: 0), the percentage of mapped reads (target value: 100%) and indels (target values: 0%).

### Genome submission

The eight bacterial genomes that had previously been assembled were deposited on the NCBI website within the BioProject PRJNA1136410. Each genome received accession numbers as follows: A0308 (CP162581), A1230 (CP162582), A1437 (CP162583-CP162584), B16120 (CP162585-CP162588), B19125 (CP162589), B21128 (CP162590-CP162591), B22129 (CP162592) and B26140 (CP162593-CP162597).

### Phylogeny of *Pseudomonas* isolates

A total of 372 *Pseudomonas* reference genomes, spanning the range from contig to complete levels, were downloaded from the NCBI_datasets v15.21.1 [50] on 11 April 2024. The eight assembled genomes were incorporated into the aforementioned references with the objective of constructing a phylogenetic tree based on the core genome. Concatenated gene alignments were performed using the cognac (R v4.2.2) [51], MAFFT v7.505 [52] and CD-HIT v4.8.1 tools [53] in order to identify shared genes in all genomes. The automatic annotations generated by Prokka were used as feature data. The multiple alignments of the selected genes were subjected to automated trimming using the software trimAl v1.4.1 with a chosen gap threshold of 10% [54]. A phylogenetic inference was conducted using maximum likelihood, employing the IQ-TREE v2.2.2.6 software [55] with model selection performed using ModelFinder [56]. A phylogenetic tree was visualized and shaped using the Interactive Tree of Life [57]. Bacterial isolates were species affiliated using the FastANI tool (v1.34) in order to compare their genomes with the closest ones found in the phylogenetic analysis [58]. A value above 95% considered that isolates belonged to the targeted reference genomes.

### Antibiotic resistance genes and their genetic environment

The search for antibiotic resistance genes was conducted on fasta files using RGI v6.0.3 with the CARD database [59], as well as ResFinder v4.5.0 [60,61] (http://genepi.food.dtu.dk/resfinder). The presence of integrons was investigated in all genomes using the Integron_finder v2.0.3 software [62]. Among mobile genetic elements, putative plasmid contigs were subjected to analysis using Plasmidfinder v2.1.6 [63]. In the event that no match was identified within the *Enterobacteriaceae* database, the DNA sequences were subjected to a comparison with NBCI-blast database [50]. ICEs were also examined using the ICEfinder website (https://bioinfo-mml.sjtu.edu.cn/ICEfinder/ICEfinder.html) in conjunction with the ICEberg 2.0 and then 3.0 databases [64]. Further investigation into the genetic architecture was conducted using the Proksee website (https://proksee.ca/) [65]. In instances where antibiotic resistance genes were located on the same genetic structure as ICE on the chromosome and plasmid, we made a nucleotide alignment between genome and plasmid sequences using unimap v0.1(r-41) [66] and then an arrow map using gggenomes v1.0.1 with ggplot2 v3.5.2 on R v4.4.2 [67,68] and Proksee.

### Focus on colistin resistance

Three out of the eight studied *Pseudomonas* exhibited resistance to colistin at varying MIC levels, with values of 16, 512 and above 1,024 µg ml^−1^ for B16120, B19125 and B22129, respectively. In order to understand the genetic origin of these phenotypes, we first searched the presence of genes coding for known two-component systems involved in LPS modifications such as PmrA/PmrB, PhoP/PhoQ and RstA/RstB. Mutations in amino acid sequences of these two-component systems were investigated using a multiple alignment analysis using Clustal Omega [69]. Multiple alignment has been visualized by Jalview [70]. Then, we searched genes coding for phosphoethanolamine transferase as *mcr*, *ept* and *cptA* to determine their intra- or extrachromosomal position and differences between low and high MIC isolates. Furthermore, we checked the presence of *arn* gene copies due to their involvement in the synthesis of *l*-Ara4N, a component of the LPS.

## Results

### Quality of assembled genomes and phylogenetic affiliation of the studied isolates

The quality control of the eight whole-genome assemblies was evaluated using the 3C criterion, which assesses contiguity, completeness and correctness (Table 2).

**Table 2.** Quality assessment of assembled genomes using the 3C criterion procedure adapted from Molina-Mora *et al*. [42]

3C criterion	Level	Metric	A0308	A1230	A1437	B16120	B19125	B21128	B22129	B26140
**Contiguity**	Contigs assembly	Contigs	1	1	2	4	1	2	1	5
		Total length	6,425,841	6,129,803	6,505,488	6,410,102	6,562,299	6,119,421	6,820,881	6,239,350
		GC (%)	58.62	58.75	58.62	59.84	59.99	58.93	60.39	58.69
		N50	6,425,841	6,129,803	6,425,841	6,364,262	6,562,299	6,021,442	6,820,881	6,006,034
		L50	1	1	1	1	1	1	1	1
		N’s per 100 kbp	0.00	0.00	0.00	0.00	0.00	0.00	0.00	0.00
**Completeness**	782 core genes (BUSCO)	Fragmented genes	1	2	1	0	0	1	0	1
		Intact genes	781	780	781	782	781	781	781	781
		Lost genes	0	0	0	0	1	0	1	0
		Completeness score (%)	99.7	99.7	99.8	100.0	99.8	99.9	99.8	99.8
	Whole-genome annotation	CDS	5832	5520	5937	5809	5871	5434	6118	5684
		Contigs	1	1	2	4	1	2	1	5
		rRNA	19	19	19	19	19	19	16	19
		tmRNA	2	1	2	1	1	1	1	1
		tRNA	73	74	73	74	67	76	68	75
**Completeness and correctness**		Mean length of CDS (bp)	955.67	963.37	951.07	963.89	979.75	976.52	984.10	950.27
	Mismatches and indels	General error rate	0.0023	0.002	0.0024	0.0028	0.0025	0.0024	0.0023	0.0024
		% of mapped reads	99.74	99.82	99.73	99.81	99.75	99.79	99.79	99.80
		% of mapped reads with insertion	0.04	0.04	0.04	0.04	0.04	0.04	0.04	0.04
		% of mapped reads with deletion	0.13	0.13	0.13	0.15	0.14	0.13	0.14	0.14

The N50 values for the eight genomes exhibited a range of 6,021,442 to 6,820,881 bp. We obtained highly continuous genomes with a single contig corresponding to the circular chromosome. Four out of eight genomes contained additional contigs ranging in size from 2,607 bp to 97,979 bp. Analysis of the 782 Pseudomonales-related core genes revealed a completeness score of the assemblies close to 100%, with a high number of intact genes found. Mapping of short reads to assemblies showed low overall error rates ranging from 0.002 to 0.0028 with a constant percentage of indels across genomes.

**Fig. 1.**
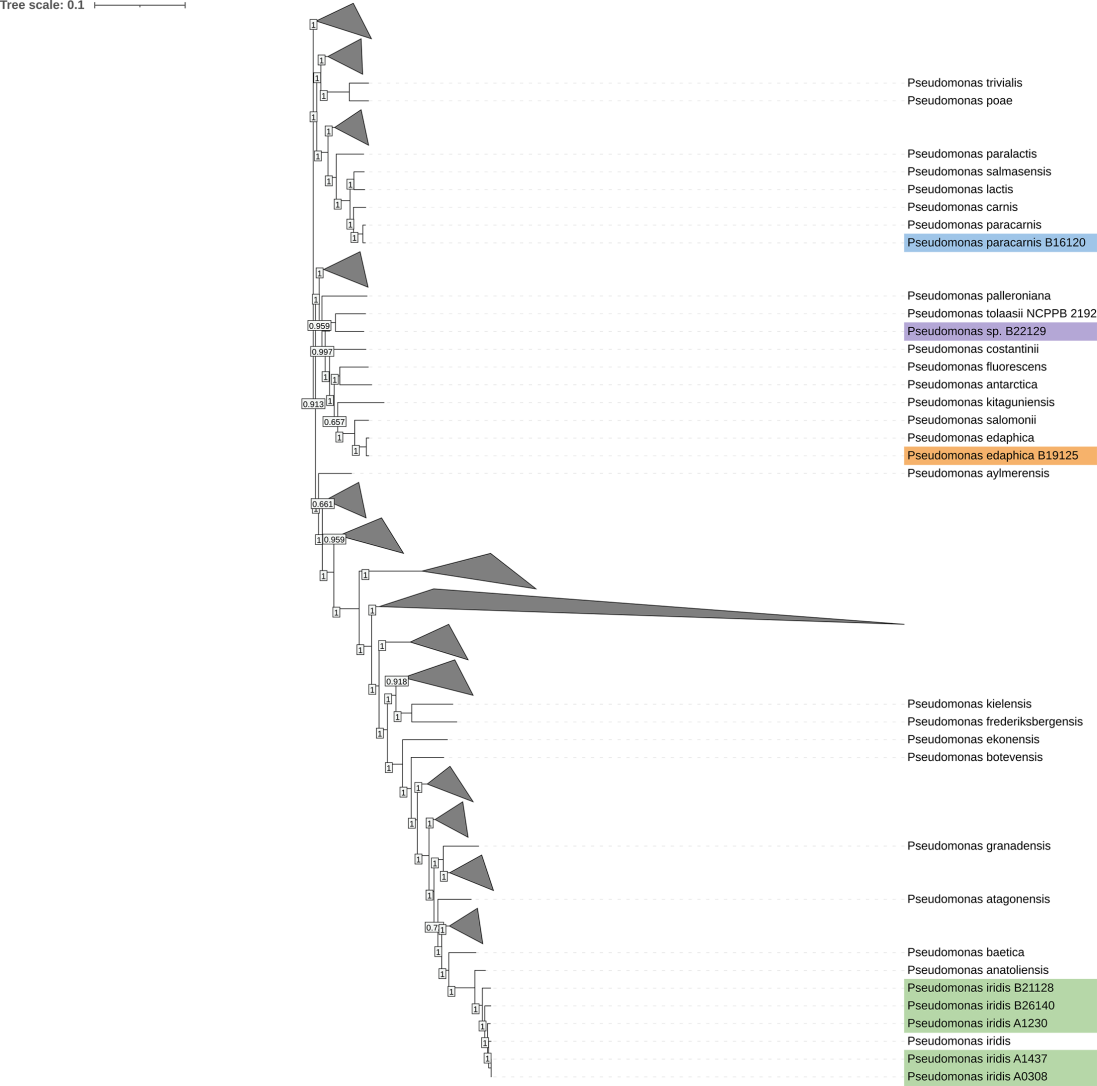
Maximum-likelihood phylogenetic tree of the eight studied *Pseudomonas* within 372 reference genomes. Studied isolates were coloured according to their species affiliation. Blue, *Pseudomonas paracarnis*; purple, unknown species close to *Pseudomonas tolaasii*; orange, *Pseudomonas edaphica*; green, *Pseudomonas iridis.* Bootstrap values are indicated for each node. Some branches have been collapsed to make the tree more visible.

An approximate maximum-likelihood phylogenetic analysis of the eight isolates coupled to the 372 *Pseudomonas* reference genomes available in the NCBI dataset was performed using 208 common genes identified by the cognac tool (Fig. 1).

This analysis showed that isolates A0308 (Average Nucleotide Identity - ANI 98.83), A1230 (ANI 98.34), A1437 (ANI 98.84), B21128 (ANI 96.17) and B26140 (ANI 96.90) belonged to the *Pseudomonas iridis* species. On the contrary, isolate B16120 (ANI 99.03) belonged to *Pseudomonas paracarnis*, and the isolate B19125 (ANI 99.01) was found as *Pseudomonas edaphica*. Considering the phylogenetic analysis, the B22129 isolate was close to *Pseudomonas tolaasii*, but the ANI value (91.61) showed that it could belong to an unidentified species within the *Pseudomonas* genus.

### Origin of high MIC phenotypes and structures harbouring resistance genes

To explain the antibiotic resistance phenotypes of the eight chosen *Pseudomonas*, the genomes were examined in more detail using Resistance Gene Identifier and ResFinder (Table 3).

**Table 3.** Antibiotic resistance genes identified in the eight *Pseudomonas* complete genomes

Black boxes: resistance gene detected in the bacterial genome and related to non-wild-type or resistance phenotype. Grey boxes: resistance genes detected in the genomes without phenotype information. White boxes: genes not detected in the genomes and no phenotype information available.

**Fig. 2.**
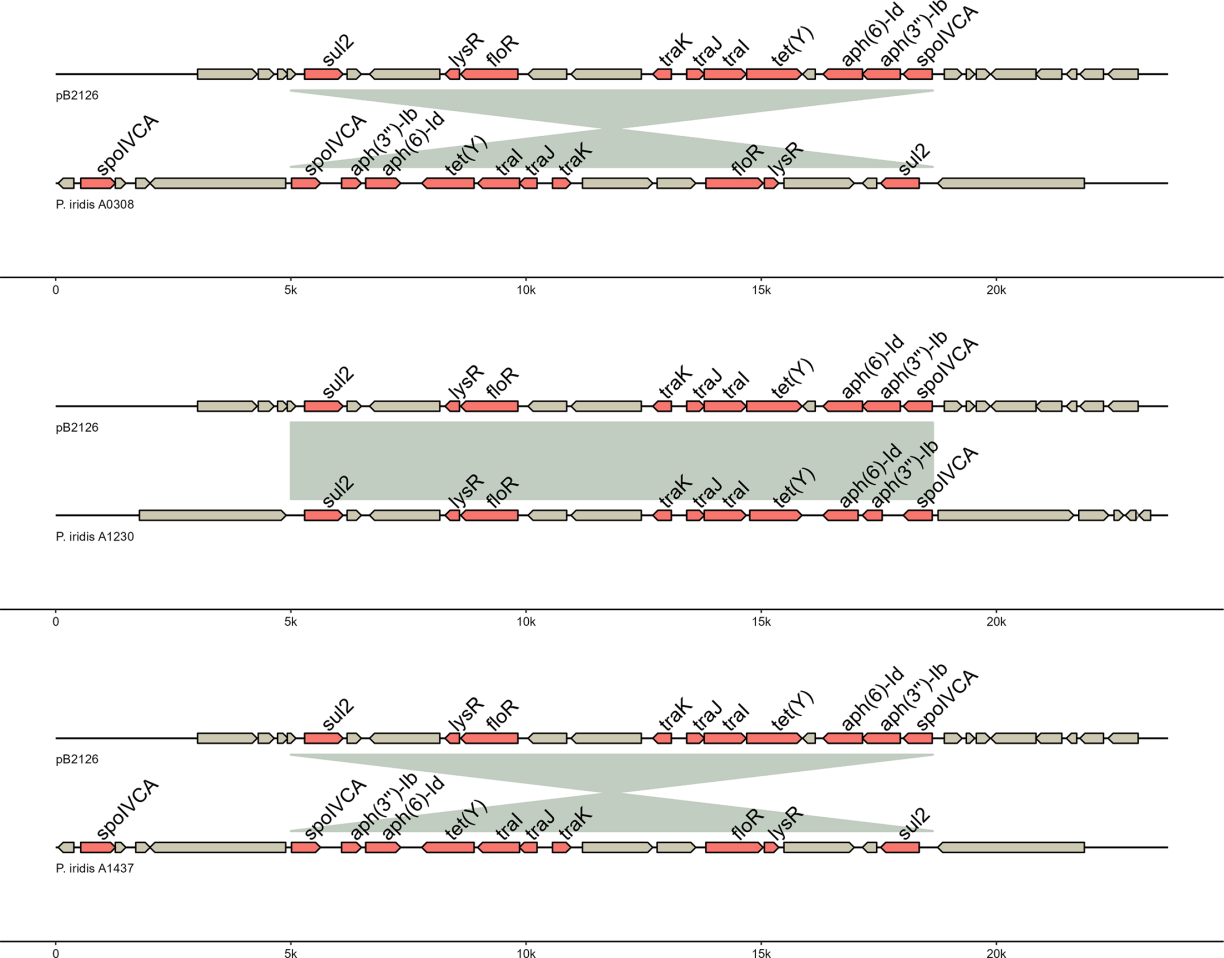
Arrow maps of genomic alignments in regions containing antibiotic resistance genes in pB2126 plasmid (upper sequence) and three *P. iridis* isolates (A0308, A1230 and A1437). In all *P. iridis* isolates, antibiotic resistance genes were located on an ICE containing *traI*, *J* and* K* genes. Automated annotation of genes (in red) was performed by Bakta. Grey areas show nucleotide similarities (>99%).

Both tools identified resistance genes with high percentages of identity (>99%) and coverage (>99%) such as *sul2*, *floR* and *tet(Y*) genes related to sulphonamide, florfenicol and oxytetracycline phenotypes previously observed in A0308, A1230, A1437, B21128 and B26140 isolates. Additional genes, in particular *aph(3″)-Ib* and *aph(6)-Id*, were found by both tools for a predicted streptomycin resistance phenotype. The *dfr* gene was not found in any genome but differences were observed in the number of *fol* gene copies within genomes (from 10 to 12). For example, all isolates with high resistance phenotypes carried four to five *folE* gene copies instead of three in susceptible bacteria. Some putative genes were only detected by the RGI tool in nudge mode but with different percentages of identity (33.71%–80.65%) or coverage (13.12%–101.38%). These genes encoding for putative efflux pumps, target modification or antibiotic inactivation were also found in all or part of the genomes studied, such as the *adeF* gene (from two to four strict hits), *soxR* (one strict hit), *vanG/W* (one strict hit), *fosA8* (one strict hit), *arnT* (one strict hit) or *ampC* (one strict hit). Among the genes detected by both tools and their associated databases, we focused on their genetic environment within the genome. Seven out of eight isolates showed the presence of Integrative Mobile Element (IME) and/or CE. Only the A1230 isolate did not harbour IME or ICE structures according to the ICEberg 2.0 or 3.0 databases. In a surprising way, some ICEs detected using ICEberg 2.0 were not detected by the 3.0. In *P. iridis* isolates (A0308, A1230 and A1437), an ICE containing all antibiotic resistance genes was found and we compared it with the pB2126 plasmid sequence harboured by other *P. iridis* isolated from another rainbow trout farm (B21128 and B26140) (Fig. 2).

Genomes of the A0308, A1230 and A1437 *P. iridis* isolates harboured ICE sequences at different regions, respectively 4,705,784–4,719,432, 3,808,239–3,821,887 and 6,089,176–6,102,824. Nucleotide alignment of these sequences with the one of pB2126 showed that antibiotic resistance genes were in the vicinity of the same genetic background (similarity >99%). Genes involved in horizontal transfer such as *traIJK* and *trbL* genes but also the presence of a transposase coding gene from the Tn3 family (Tn5393) were also detected in the regions studied with 89.29% protein identity between A0308 (gene AB4P92_21885 : 4,702,788–4,705,673), A1437 (gene AB4P95_RS28325 : 6,086,180–6,089,065) and A1230 (gene AB4P97_RS17840 : 3,821,999–3,824,884) isolates (not annotated on Fig. 2). A spoIVCA coding gene was also found in all genomic and plasmid sequences at a putative insertion site.

**Fig. 3.**
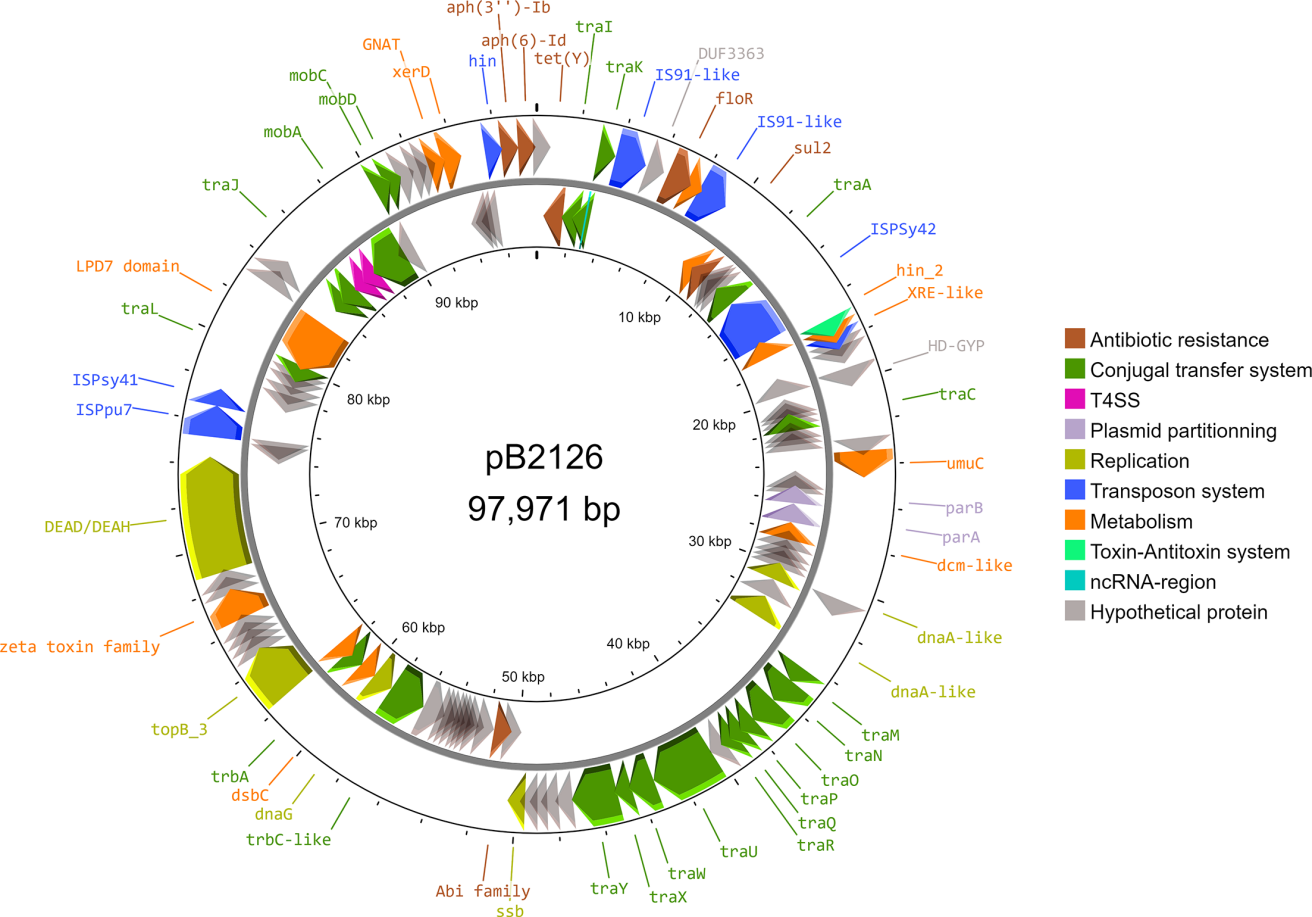
Arrow map of the plasmid pB2126 found in B21128 and B26140 isolates. Antibiotic resistance and other relevant genes were coloured according to the legend presented in the figure.

Small contigs found after the assembly step were assumed to be plasmids, one of which was found to be linear during the Trycycler process (B26140; 39,001 bp). No hits were found with the Plasmidfinder tool, but alignment with the NCBI database showed hits with named or unnamed plasmids. In this study, we focused our analyses on those carrying antibiotic resistance genes. Both *P. iridis* isolates B21128 and B26140 contained similar 97 kb DNA sequences with high nucleotide similarity (99.98%) and coverage (100%). Nucleotide sequence comparison with the NCBI database showed high identity percentages ranging from 92.61% to 99.92% and covering from 73% to 78% of the three identified plasmids (GenBank accession numbers CP146345.1, JX891462.1 and CP141834.1). All CDS identified by the Bakta and Prokka databases were compared with the NCBI protein database to provide an accurate annotation of the putative plasmid newly named pB2126 (Fig. 3). Further examination of these sequences revealed the presence of the antibiotic resistance genes *tet(Y*), *floR* and *sul2*, which are associated with phenotypic characteristics of bacteria (Fig. 3, Table 1). In addition, genes involved in conjugal transfer were identified, such as *tra*, *mob* and *trb*. Several insertion sequences were found, especially genes close to IS91, ISPSy41, ISPSy42 and ISPpu7. As mentioned above, the pB2126 region harbouring resistance genes showed a high percentage of nucleotide identity (99.97%) with the ICE found in A0308, A1230 and A1437 isolates, which covered only 13% of the plasmid sequence.

### Genetic differences between sensitive and resistant phenotypes to colistin

**Fig. 4.**
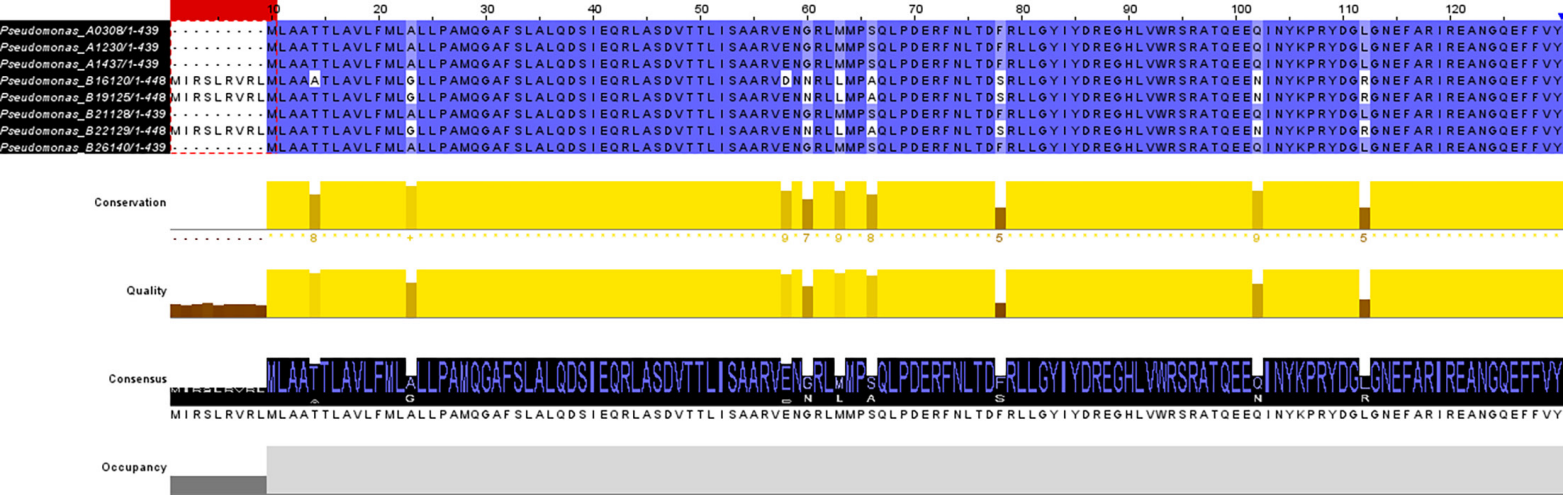
Alignment of amino acids of the PhoQ protein of the eight *Pseudomonas* isolates. A nine-amino-acid insertion was detected at the protein N-terminal extremity for the three isolates showing high MIC values against colistin (red box).

Due to the use of colistin as an antibiotic of last resort, mobile genes encoding phosphoethanolamine transferase called *mcr* were examined in all isolates, especially in those with non-wild-type phenotypes (Table 1). No such genes were found in all genomes, but we identified some differences between isolates with low and high MIC values. First, we focused on the known two-component regulatory systems in *Enterobacteriaceae*, namely, *pmrA*/*pmrB* and *phoP*/*phoQ*. Within all genome isolates, no *pmrA*/*pmrB* system was detected using automated annotation by Prokka and Bakta. However, phoP/phoQ genes were found in all genomes with wide differences between isolates showing low MIC values (2 µg ml^−1^) to colistin and those with high values (from 16 µg ml^−1^ to upper than 1,024 µg ml^−1^). Analysis of amino acid sequences of both *phoQ* and *phoP* genes highlighted a wide number of mutations in the three resistant isolates (Table 4). Sequence of the PhoQ protein also showed an insertion of 9 amino acids at the N-terminal extremity in isolates with high MIC values against colistin (Fig. 4). Additionally to these findings, we also identified the two-component regulatory system *rstA*/*rstB* in all genomes. Comparisons of amino acid mutations did not show relevant differences between the two categories of isolates (low and high MIC values). Then, we searched genes that can be activated by these two-component regulatory systems and highlighted a wide diversity of genes in isolates with high MIC values (Table 5). Thus, *arn* genes involved in l-Ara4N addition to the LPSs were found in multiple copies for some genes, especially *arnT*. Analysis of genes coding for phosphoethanolamine transferase also showed difference with the presence of *eptA* (two copies) and *eptC* (one copy) genes in low MIC value isolates instead of the *cptA* (one copy) gene in high MIC isolates.

**Table 4.** Comparison of mutations in the two-component system PhoP/PhoQ found in the eight *Pseudomonas* isolates

Isolate ID	PhoP	PhoQ
A0308	–	R330Q
A1230	–	–
A1437	–	R330Q
B16120	Q17F, E22D, S29A, F45H, V49I, S68T, G69Q, G70T, D109E, L112M, P170A, T225R	T14A, A23G, E58D, G60N, M63L, S66A, Q102N, L112R, K135R, E156Q, T157L, A179T, I183L, Q190K, Q194R, Q197H, E201Q, T204G, T211S, Q213E, L230H, Q234E, S237A, K269E, D270E, V271R, D272S, S289G, R310E, L313V, D312E, R330T, E337Q, H338A, Y340E, Q345K, M352L, G365K, E366Q, V371L, R372V, S374N, T376D, V378I, V382I, E399Q, N426H, K428R, A430T, P435A, M436L, V448L
B19125	Q17F, E22D, S29A, F45H, V49I, G69Q, G70A, D109E, L112M, P170A, T225R	A23G, G60N, M63L, S66A, Q102N, L112R, E156Q, T157L, R163Q, A179T, I183L, Q190K, Q194R, E201Q, T204G, Q213E, L230H, Q234E, S237A, K269E, D270E, V271R, G272S, S289G, R310E, R330T, D334E, E337P, H338E, Y340D, Q345K, M352L, G365S, V371L, R372T, S374N, T376E, V378T, V382I, E399Q, N426S, K428R, A430T, M436L, V448A
B21128	–	A430M
B22129	Q17M, E22D, S29A, F45H, V49I, G69Q, G70A, D109E, L112M, P170A, T225R	A23G, G60N, M63L, S66A, Q102N, L112R, E156Q, T157L, R163Q, A179T, I183L, Q194R, T204G, T211S, Q213E, L230H, Q234E, S237A, K269E, V271R, S289G, R310E, E337P, H338D, Y340D, Q345K, M352L, L364V, L364V, G365R, S370R, V371L, R372V, T376E, V378T, V382I, E399Q, N426S, K428R, A430T, M436L
B26140	–	T206A

**Table 5.** Genomic locations (bp) of known LPS-modifying genes involved in colistin resistance

Isolate ID	*arn*A	*arnB*	*arnC*	*arnD*	*arnE*	*arnF*	*arnT*	*eptA*	*eptC*	*cptA*
A0308	nd	1,642,079–1,643,242	nd	nd	nd	nd	nd	1,790,026–1,791,675 /3,396,372–3,398,057	421,133–422,881	nd
A1230	nd	697,634–698,791	nd	nd	nd	nd	nd	554,385–556,034/5,085,803–5,087,488	1,880,788–1,882,536	nd
A1437	nd	3,025,471–3,026,634	nd	nd	nd	nd	nd	3,173,418– 3,175,067/4,779,764– 4,781,449	1,804,525–1,806,273	nd
B16120	**3,112,266–3,114,257**	nd	**3,111,235–3,112,269**	**3,114,257–3,115,141**	1,386,601– 1,387,005/**3,116,790–3,117,137**	1,386,224– 1,386,604/**3,117,134–3,117,547**	**3,115,138–** **3,116,793**/3,984,658– 3,986,313/3,986,342–3,987,859/5,318,237– 5,319,670	nd	nd	3,550,693–3,552,429
B19125	**3,670,148–3,672,139**	734,616–735,758/773,333–774,550	nd	**3,669,264–3,670,148**	774,547–774,963/774,960–775,316 / 2,196,962–2,197,300	**3,666,858–3,667,271**	1,361,841–1,363,274/2,741,125– 2,742,642/2,742,669– 2,744,327/**3,667,268–** **3,667,615/3,667,612–3,669,267**	nd	nd	3,339,831–3,341,573
B21128	nd	5,549,294–5,550,457	5,707,521–5,708,528	nd	nd	nd	nd	1,248,327–1,250,027 / 5,698,090–5,699,739	4,413,074–4,414,822	nd
B22129	**4,741,176–4,743,167**	1,715,881–1,717,023/1,755,367–1,756,587	**4,743,164–4,744,198**	**4,740,292–4,741,176**	1,756,587–1,757,000/1,756,997–1,757,353 / **4,738,296–4,738,643**	**4,737,886–4,738,299**	2,340,464–2,341,897/3,770,440–3,771,957/3,771,985–3,773,640/**4,738,640–4,740,295**	–	–	5,196,598–5,198,340
B26140	nd	153,665–154,828	nd	nd	nd	nd	nd	4,375,046–4,376,746 / 5,998,077–5,999,726	1,189,097–1,190,845	nd

nd: gene not detected within the genome using Prokka and Bakta annotation tools. In bold, genes found in the same genomic region.

## Discussion

To ensure that genomic analyses are consistent with the biological function of the sequenced bacteria, it is imperative to check the quality of the assembled genomes. Although there is no harmonized procedure yet, several metrics have been proposed for the quality control assessment [42,71]. In our study, eight *Pseudomonas* whole genomes with high MIC values against different antibiotics were assembled and their quality was checked using the 3C criterion (contiguity, completeness and correctness) adapted from a previous work [42]. The *Pseudomonas* genomes showed a high contiguity based on the metrics used and the sizes of genomes (6.02–6.82 Mbp) were consistent with those previously studied [72]. Furthermore, the very low number of fragmented or lost genes compared to the 782 core genes of the *Pseudomonales* database shows that the eight genome assemblies and annotations produced in the present study are of high quality and with optimal completeness. Correctness was the limitation of our quality control assessment due to the use of only one strategy based on the alignment of short reads on polished genomes. However, the percentage of mapped reads was higher than 99% and low indel errors were low compared to previous works on different bacterial genomes [42,73].

According to our approximate maximum-likelihood phylogenetic analysis, the *Pseudomonas* isolates included here belonged to different species with five genomes in close proximity and related to the same species (*P. iridis*). Three other genomes were found to belong to different species, suggesting that the isolates analysed in our study are related to four separate species. Our isolates have already been found associated with fish [74,75], rhizosphere [76] or even meat [77] for *P. paracarnis*. This result was surprising but probably due to the recent discovery of this species, highlighting that our study could be the first to describe it associated with rainbow trout fillets. Based on our previous results on these isolates identified by MALDI-TOF technology [16], all belonged to the *P. fluorescens* group but analysis of the 208 common CDS with reference genomes showed different phylogenetic affiliations. The recent discovery of some species (*P. iridis* and *P. anatoliensis*) and the methodological approach chosen (based on the selection of 208 common CDS by the Cognac tool) could explain the difference between the phylogeny presented in this article and those already published using other genes [78,80]. We also noticed that isolates A0308 and A1437 had the same chromosome size and were very close in terms of phylogeny. The main difference seemed to be the acquisition of a putative plasmid by A1437.

Non-wild-type or resistant phenotypes to oxytetracycline, florfenicol and sulphonamides were explained by the genetic content of the *Pseudomonas* previously isolated from rainbow trout fillets from two different farms (A and B) located along the same river. The trimethoprim resistance phenotypes were not explained by the presence of *dfr* genes in the genomes. However, we found that the number of *folE* copies was higher in resistant isolates. This gene is known to encode a GTP hydrolase which catalyses the first step of the tetrahydrofolate biosynthetic pathway [81]. We hypothesized that one or two additional *folE* genes in the genomes could improve bacterial metabolism and make them resistant to trimethoprim. A previous study on *Burkholderia pseudomallei* has also highlighted that the presence of the *folE* gene at several loci was unable to conclude whether it could explain trimethoprim resistance [82]. Genes encoding aminoglycoside resistance have also been detected by Resistance Gene Identifier and ResFinder tools. No phenotyping studies were performed for these resistances, but several studies have already identified these genes as part of the resistome within the *Pseudomonas* species [83,84]. Although CARD and ResFinder databases had different error rates [85], they both identified well genes explaining the phenotypes of the studied isolates. Additional genes were only detected by the RGI tool in nudge mode, several of which are likely to be involved in the efflux of several classes of antibiotics, reinforcing the high MIC values assessed in our previous work. We also identified that *tet(Y*), *floR*, *sul2*, *aph(3″)-Ib* and *aph(6)-Id* genes were located on chromosome or plasmid depending on the isolates with high nucleotide similarity. This result suggests that ICE harbouring these resistance genes can be integrated on different genetic structures within the same species (*P. iridis*). It is noteworthy that these genes were found in the A1230 but surprisingly not on an ICE according to the ICEberg 3.0 database. Contrary to other identified isolates, this genetic region was completely reversed. It remains to be seen why this genetic region is not detected by the current 3.0 database [86]. In addition, differences in phenotypic expression, especially for oxytetracycline and trimethoprim-sulfamethoxazole association, could be explained by this genomic inversion between A0308/A1437 and A1230 isolates, as already studied in *E. coli* [87]. The location of the antibiotic resistance genes on two different genetic elements could indicate a genetic event in which the ICE could be integrated in the pB2126 plasmid or chromosome within the same *P. iridis* species isolated from rainbow trout reared in different farms along the same river. This event was probably mediated by prophage insertion and/or transposition events due to integrase and transposase genes found in the ICE but not in the plasmid sequence, highlighting the importance of these two mobile genetic elements in antibiotic resistance evolution [88].

Colistin is an antibiotic of last resort in human medicine and its resistance mechanisms have been reviewed [20]. In our study, we demonstrated that the pmrA/pmrB regulatory system was missing in all genomes (low or high MIC values). However, the PhoP/PhoQ system was detected with several mutations and a seven-amino acid insertion between low and high MIC *Pseudomonas* isolates. As previously highlighted in *Klebsiella pneumoniae*, this two-component regulatory system could directly activate the arn operon [18]. Interestingly, we found another two-regulatory component system (RstA/RstB) involved in the expression of colistin resistance in *Vibrio* [19]. Adding to these findings, we found a wide diversity of *arn* gene, especially *arnT* highlighted in our research of antibiotic resistance genes using RGI tool and a *cptA* gene in high MIC isolates. To better understand these phenotypes, a deeper investigation should be followed to establish the link between genotype and phenotype in low and high MIC *Pseudomonas* species.

Finally, we strengthened the knowledge of environmental *Pseudomonas* isolated from rainbow trout fillets which can be reservoirs of antibiotic resistance genes and responsible for non-wild-type or resistant phenotypes. The most impactful outcome of our study is the relationship made in phylogenetically closed species located along the same river but isolated from different rainbow trout farms, which showed that the same resistance genes can be harboured on different genetic elements. Our findings provided scientific knowledge on antibiotic resistance dynamics within the *P. iridis* species (ICE located on chromosome or on plasmid). Further studies must be performed to assess if this ICE harbouring several antibiotic resistance genes could be spread out from chromosome to chromosome or plasmid to chromosome in other *Pseudomonas* species found in aquaculture environments. It would help to improve the One Health surveillance of antibiotic resistance between the environment, humans and animals.

## Supplementary material

10.1099/acmi.0.001029.v3 Uncited Supplementary Material 1.

## Abbreviations

ICE: integrative conjugative element
IME: integrative mobile element
