# Supplementary material for: Genomic screening of eight antibiotic-resistant Pseudomonas isolated from rainbow trout (Oncorhynchus mykiss)
Source: Access Microbiol. 2026 Feb 11;8(2):001029.v3. doi: 10.1099/acmi.0.001029.v3 (PMC12893666; doi:10.1099/acmi.0.001029.v3)

A03RPs3-08 – clustering tree

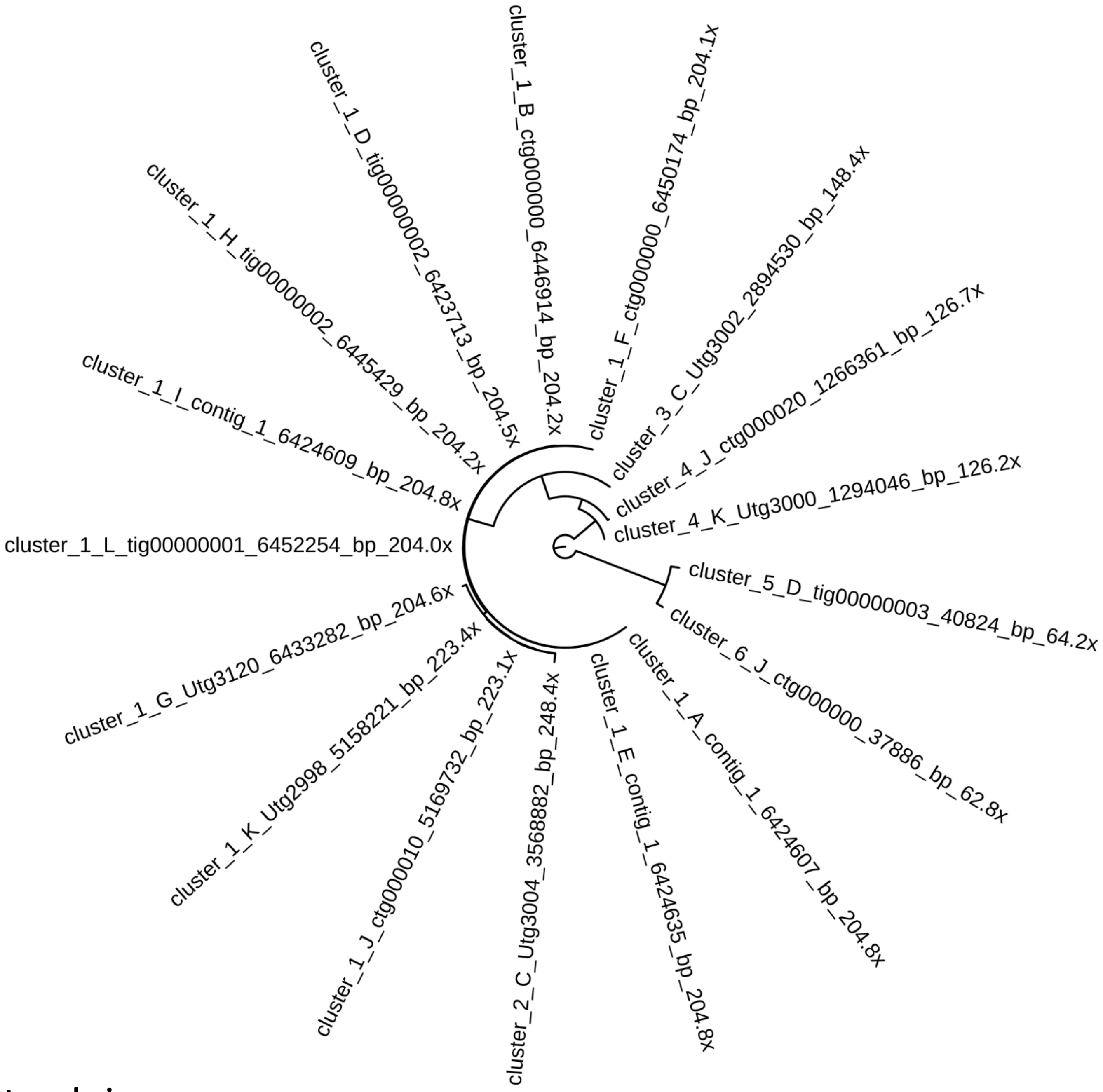

Clusters choice

- **Cluster 1 (chromosome?)**
  - contig K-Utg2998 and J_ctg000010 were removed due to their low size (5.1Mb < 6.4Mb)

- **Clusters 2, 3, 4, 5 and 6**
  - all contigs were removed due to their low size (Cluster 2) or their weak representation in the different assemblers (Clusters 3, 4, 5 and 6)

Reconcile step

❑ **CLUSTER 1 (Chromosome) – 9 contigs**

- **Contig B-ctg000000 was removed due to unability to circularize to other contigs**
  → 8 contigs remaining

- **Contig G_Utg3120 was removed due to unability to circularize to other contigs**
  → 7 contigs remaining

A12RPs2-30 – clustering tree

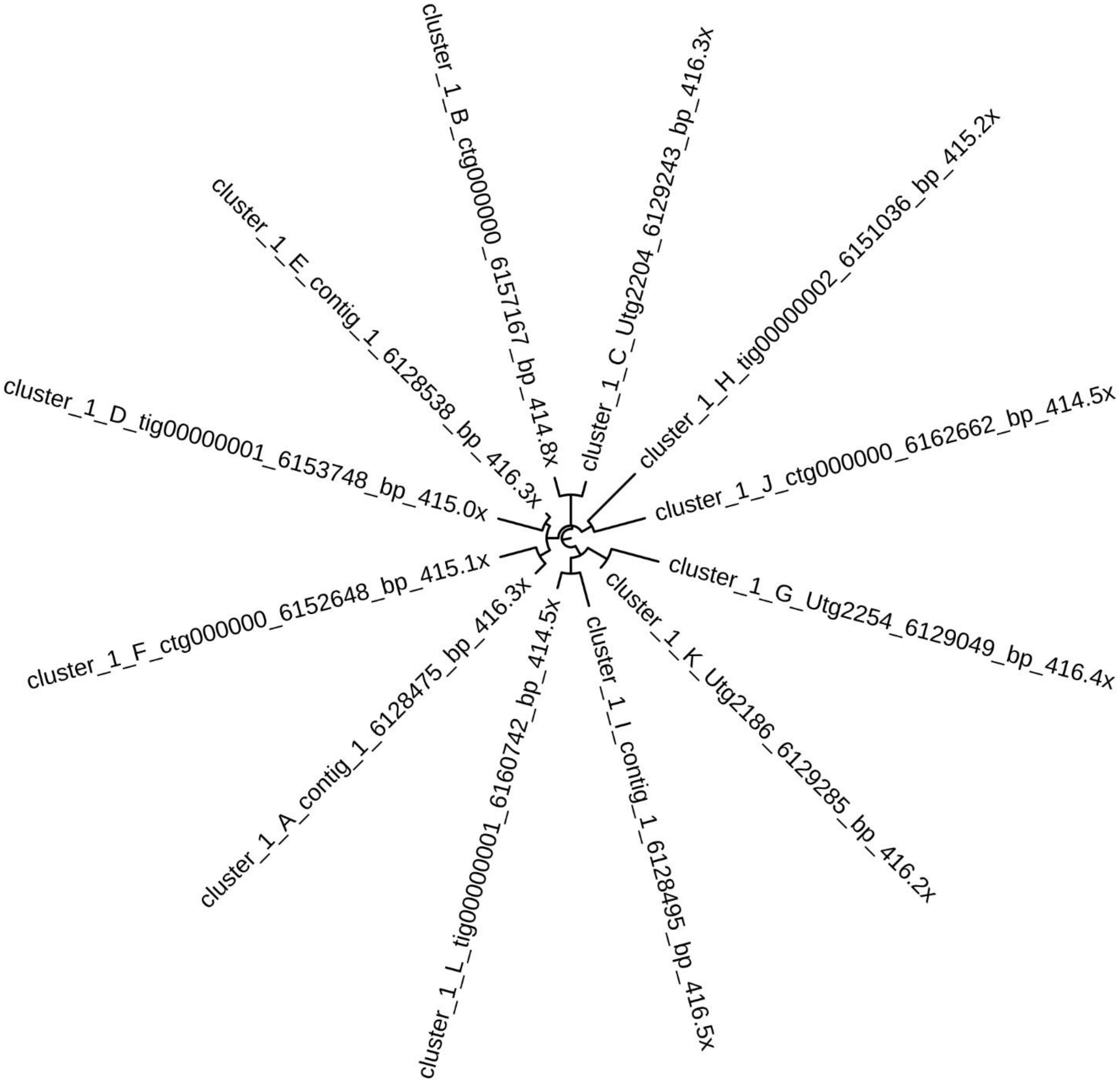

Clusters choice

- **Cluster 1 (chromosome?)**
  - All contigs were chosen

Reconcile step

❑ **CLUSTER 1 (Chromosome) – 12 contigs**

- **Contig B-ctg000000 was removed due to unability to circularize to other contigs**
  → 11 contigs remaining

- **Contig F-ctg000000 was removed due to unability to circularize to other contigs**
  → 10 contigs remaining

- **Worst-1Kb identity lower than 85%**
  - Contig K_Utg2186 (75.3% - 75.4%) was removed
    → 9 contigs remaining

A14RPs3-37 – clustering tree

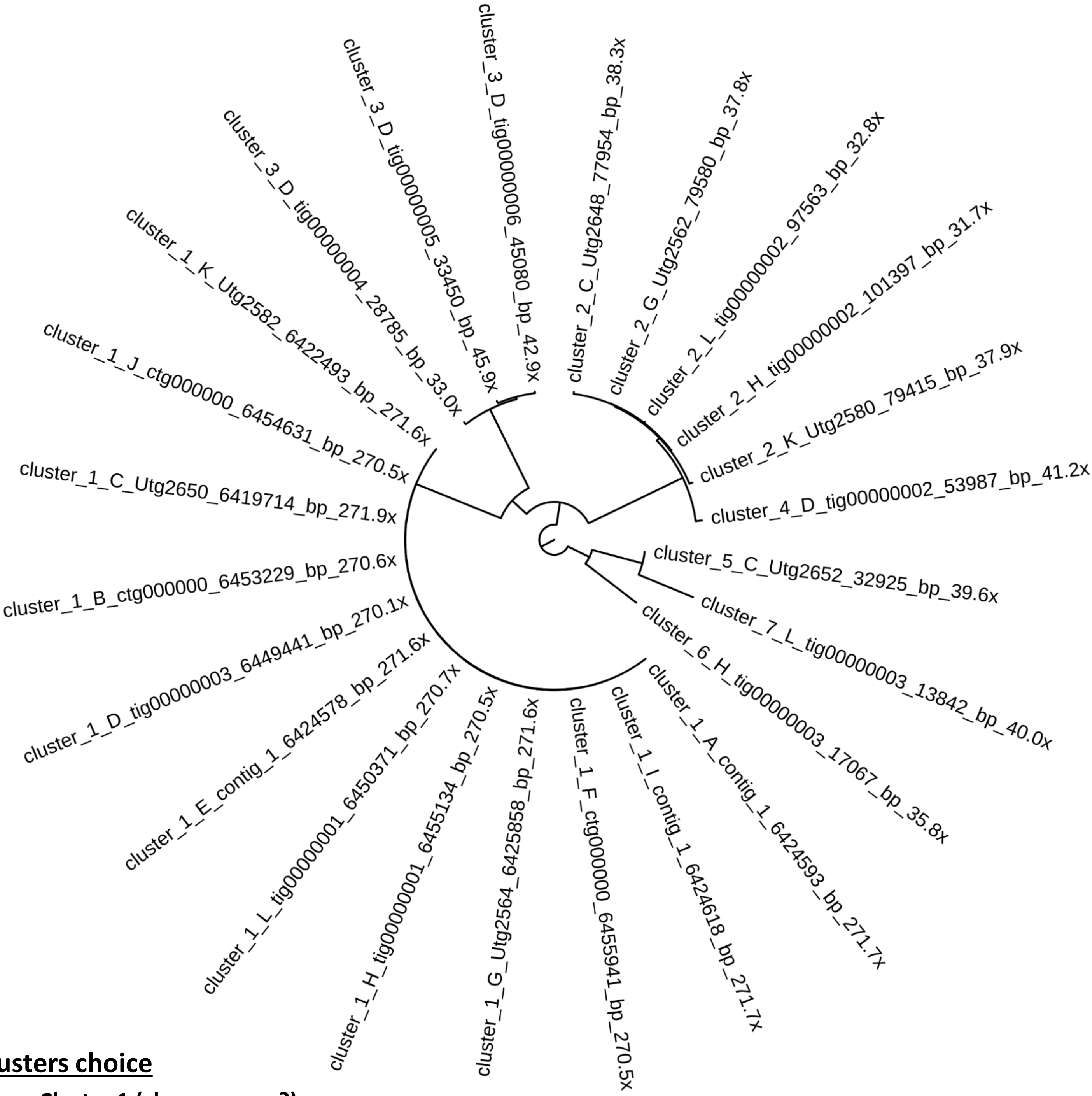

Clusters choice

- **Cluster 1 (chromosome?)**
  - All contigs were chosen
- **Cluster 2 (plasmid?)**
  - Five contigs were chosen even if they were provided by only two assemblers (Raven and Canu)
- **Clusters 3, 4, 5, 6 and 7**
  - Contigs were not chosen due to their weak representation with one or two assemblers

Reconcile step

❑ **CLUSTER 1 (Chromosome) – 12 contigs**

- **Contig C_Utg2650 was removed due to unability to circularize to other contigs**
  → 11 contigs remaining
- **Contig K_Utg2582 was removed due to unability to circularize to other contigs**
  → 10 contigs remaining
- **Worst-1Kb identity lower than 85%**
  - Contig G_Utg2564 (65.2%) was removed
    → 9 contigs remaining

A14RPs3-37 – clustering tree

Reconcile step

❑ **CLUSTER 2 (79Kb plasmid) – 5 contigs**

- **Contigs H_tig00000002 and L_tig00000002 was trimmed manually due to their long size**

- **Contig C_Utg2648 was removed due to unability to circularize to other contigs**

  → 4 contigs remaining

- **Worst-1Kb identity lower than 85%**
  - Contig K_Utg2580 (65.2%) was removed

    → 3 contigs remaining

→ **BLAST sequences belonged to a plasmid isolated from *Pseudomonas* species**

→ **It seems that it is a circular DNA based on dotplots**

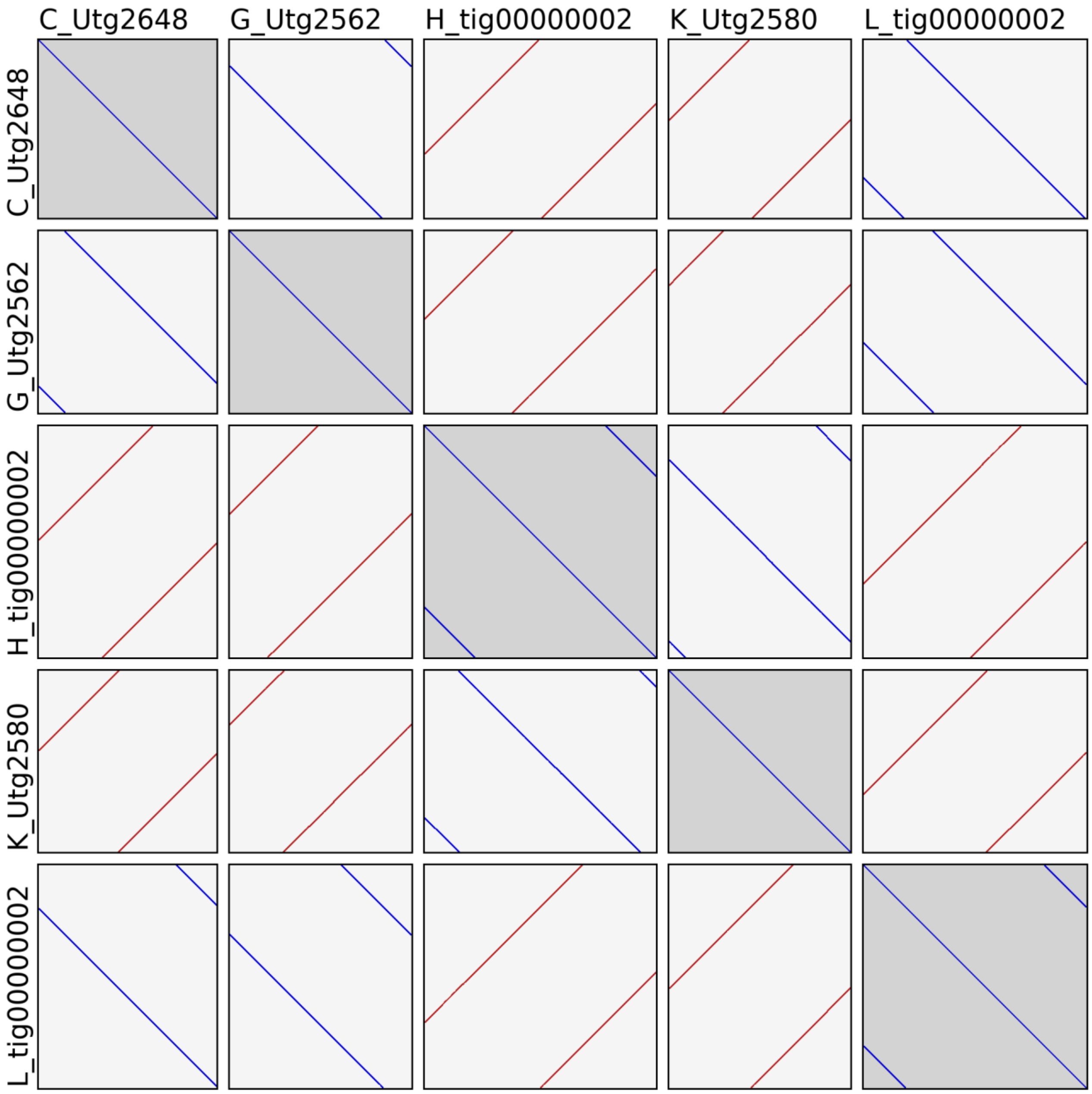

B16FPs1-120 – clustering tree

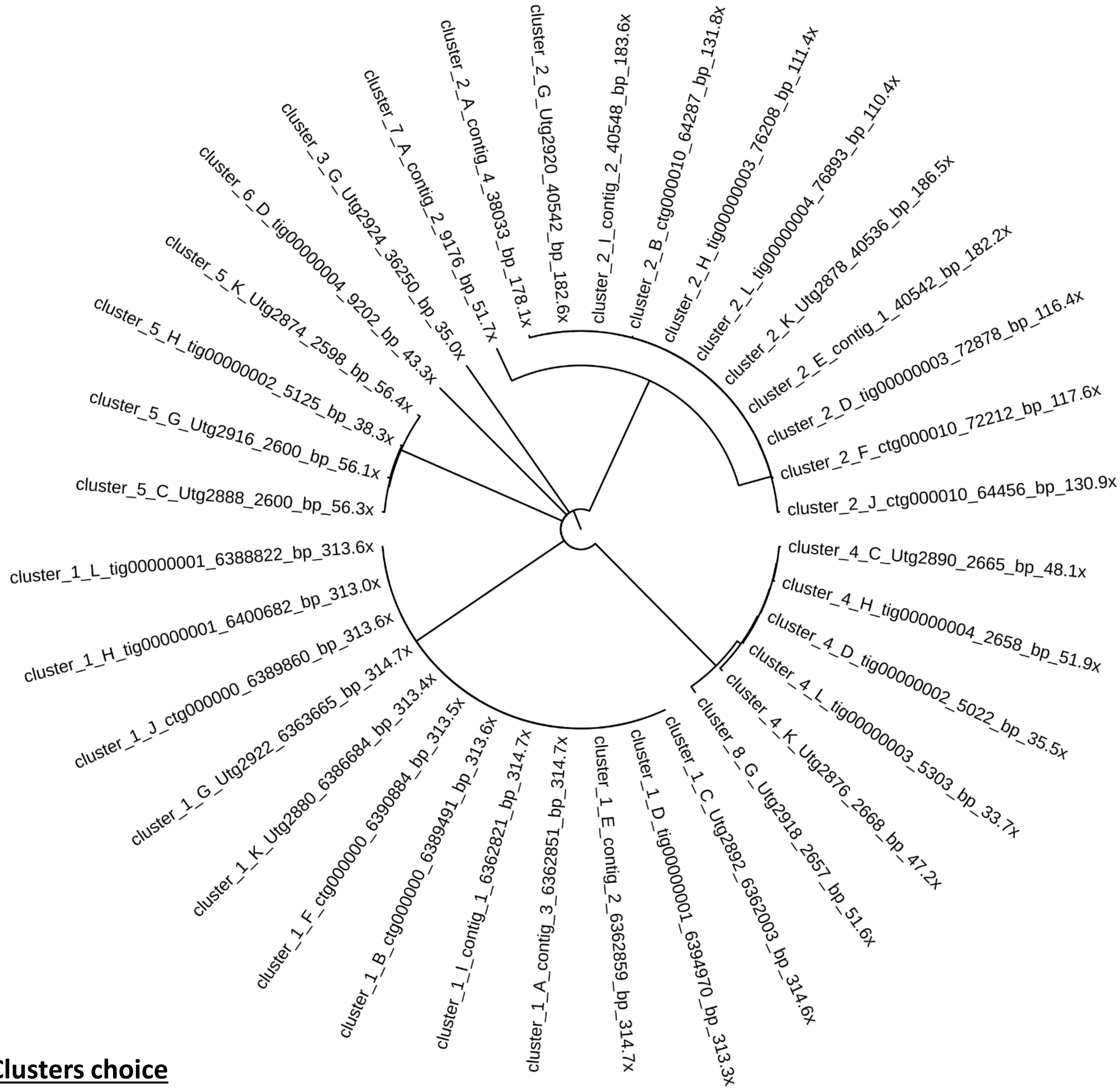

Clusters choice

- **Cluster 1 (chromosome?)**
  - All contigs were chosen
- **Clusters 2, 4 and 8 (plasmids?)**
  - All contigs were chosen
- **Cluster 5**
  - Only 4 contigs found by two out of four assemblers -> removed
- **Clusters 3, 6 and 7**
  - Each contig was removed due to their weak representation

Reconcile step

❑ **CLUSTER 1 (Chromosome) – 12 contigs**

- **Contig F_ctg000000 was removed due to unability to circularize to other contigs**

  → 11 contigs remaining

- **Worst-1Kb identity lower than 85%**
  - Contigs C_Utg2892, G-Utg2922 and K-Utg2880 were removed

    → 8 contigs remaining

B16FPs1-120 – clustering tree

Reconcile step

- ❑ **CLUSTER 2 (40Kb plasmid) – 11 contigs**
  - **Contigs B, D, J, L and M were trimmed manually**
  - **Contig A_contig_4 was removed due to unability to circularize to other contigs**
    → 10 contigs remaining

→ It seems that it is a circular DNA based on dotplots

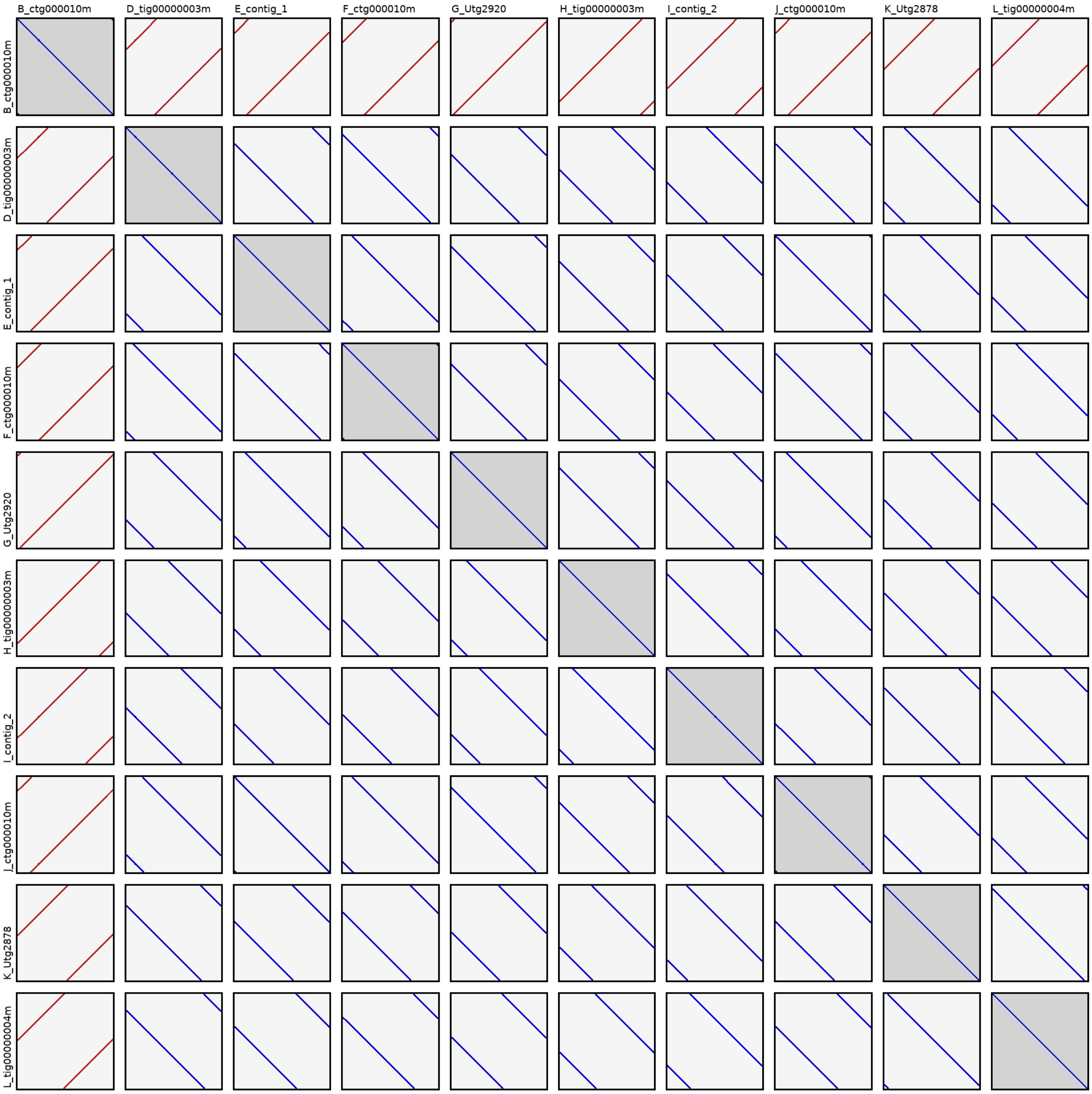

B16FPs1-120 – clustering tree

Reconcile step

❑ **CLUSTER 4 (2.6Kb plasmid) – 5 contigs**

- **Cluster_8 G_Utg2918 was added in cluster 4 due to its proximity**
  → 6 contigs remaining

- **Contigs D and L were trimmed manually**

  → **It seems that it is a circular DNA based on dotplots**

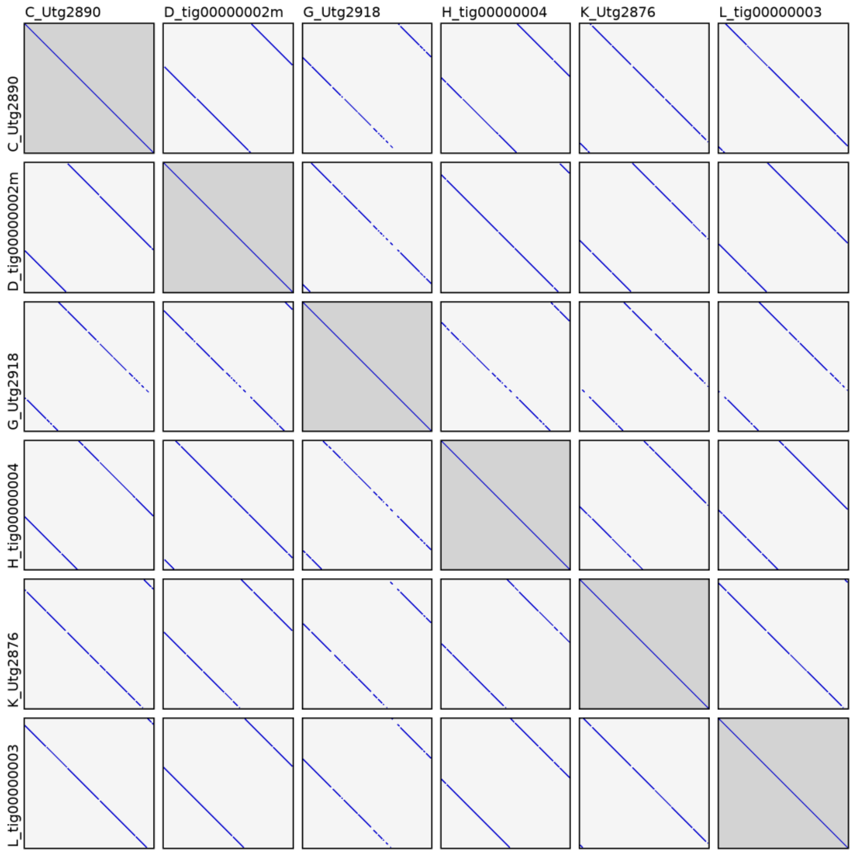

❑ **CLUSTER 5 (2.6Kb plasmid) – 4 contigs**

- **All contigs were included**

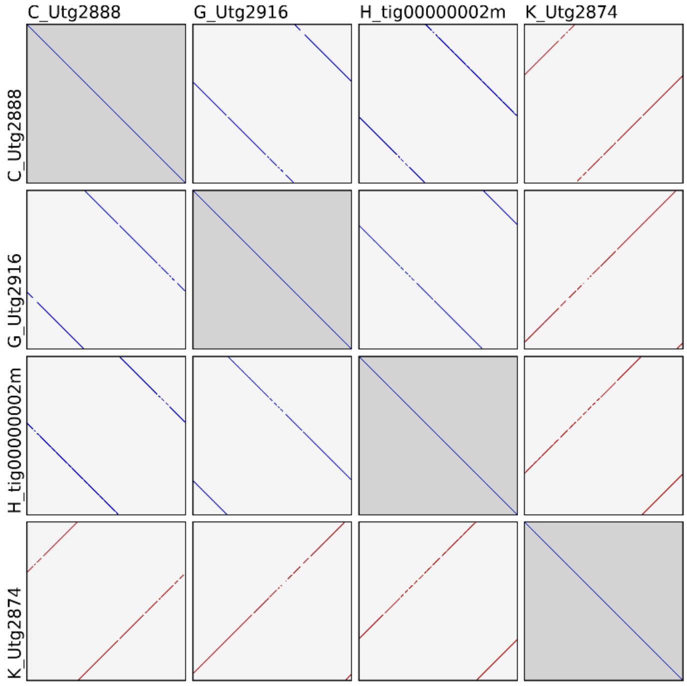

→ **It seems that it is a circular DNA based on dotplots**

B19FPs3-125 – clustering tree

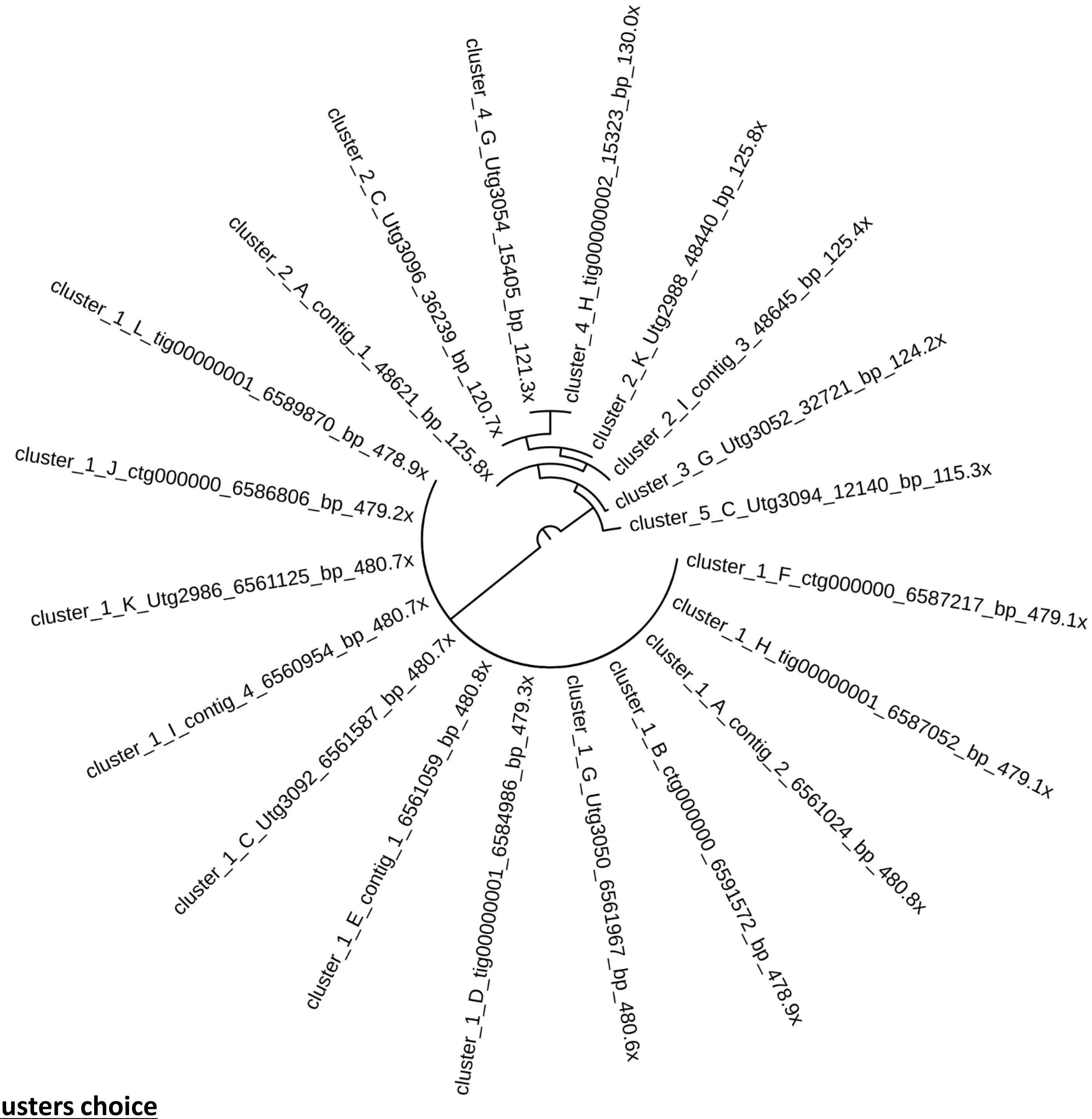

Clusters choice

- **Cluster 1 (chromosome?)**
  - All contigs were chosen
- **Cluster 2, 3, 4 and 5**
  - High heterogeneity with a weak representation -> removed

Reconcile step

❑ **CLUSTER 1 (Chromosome) – 12 contigs**

- **Contig F_ctg000000 was removed due to unability to circularize to other contigs**
  → 11 contigs remaining
- **Contig I_contig_4 was removed due to unability to circularize to other contigs**
  → 10 contigs remaining
- **Contig J_ctg000000 was removed due to unability to circularize to other contigs**
  → 9 contigs remaining
- **Contig B_ctg000000 was removed due to unability to circularize to other contigs**
  → 8 contigs remaining

B19FPs3-125 – clustering tree

Reconcile step

- ❑ **CLUSTER 1 (Chromosome) – 12 contigs**
  - **Worst-1Kb identity lower than 85%**
    - Contig G_Utg3050 (75.5%) was removed
      → 7 contigs remaining
  - **Worst-1Kb identity lower than 85%**
    - Contig K_Utg2986 (55.6%) was removed
      → 6 contigs remaining

B21FPs3-128 – clustering tree

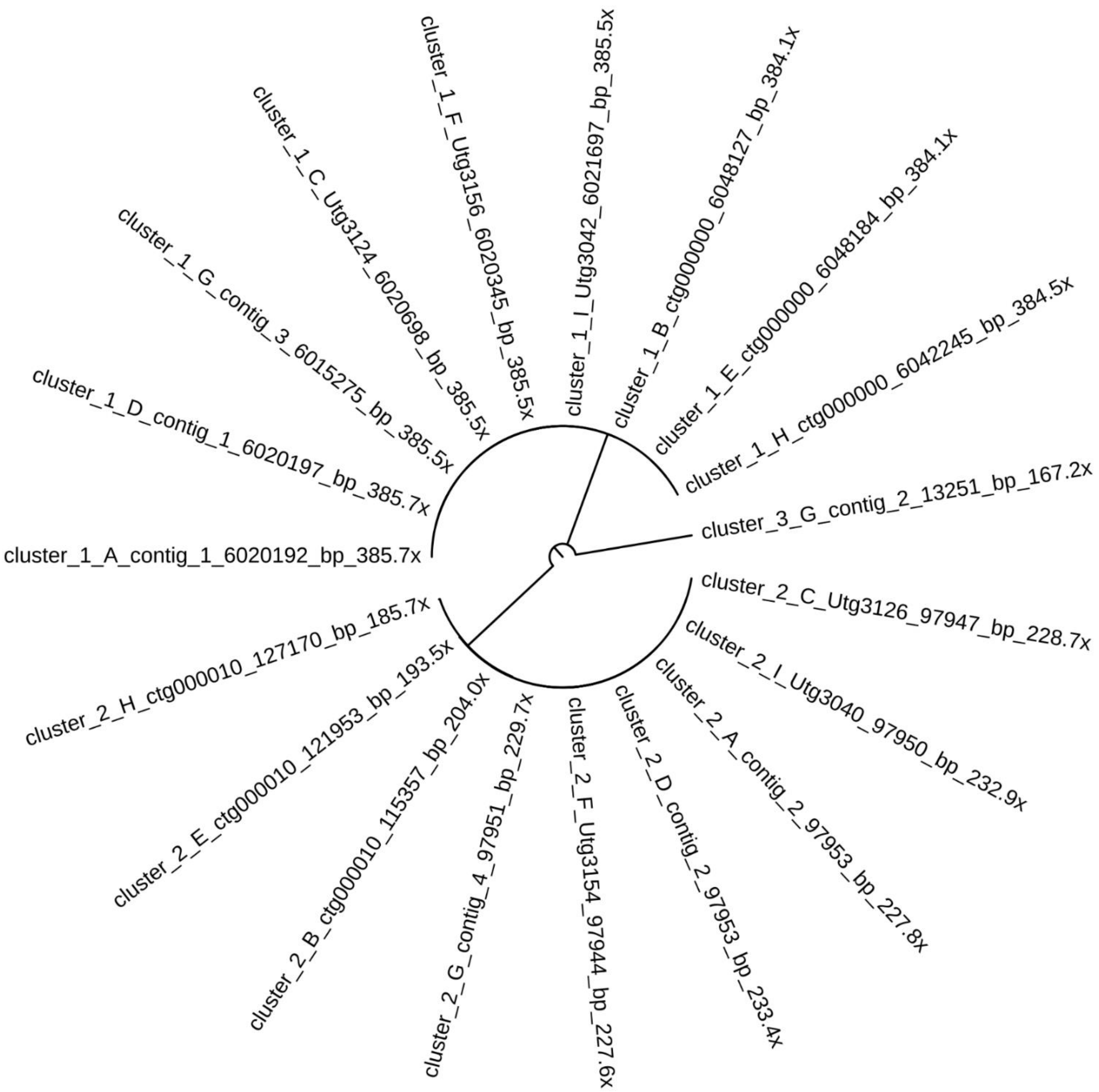

Clusters choice

- **Cluster 1 (chromosome?)**
  - All contigs were chosen
- **Cluster 2 (plasmid?)**
  - All contigs were chosen with manual trimming for contigs B, E and H
- **Cluster 3**
  - Only one contig → removed

Reconcile step

❑ **CLUSTER 1 (Chromosome) – 9 contigs**

- **Contig B_ctg000000 was removed due to unability to circularize to other contigs**
  → 8 contigs remaining
- **Contig G_contig_3 was removed due to unability to circularize to other contigs**
  → 7 contigs remaining
- **Worst-1Kb identity lower than 85%**
  - Contigs F_Utg3156 (22.4% - 23.9%) and I_Utg3042 (14.0% - 14.1%) were removed
    → 5 contigs remaining

B21FPs3-128 – clustering tree

Reconcile step

❑ **CLUSTER 2 (97Kb plasmid) – 8 contigs**

- **Contig B_ctg000010 was removed due to unability to circularize to other contigs**
  → 7 contigs remaining

→ **It seems that it is a circular DNA based on dotplots**

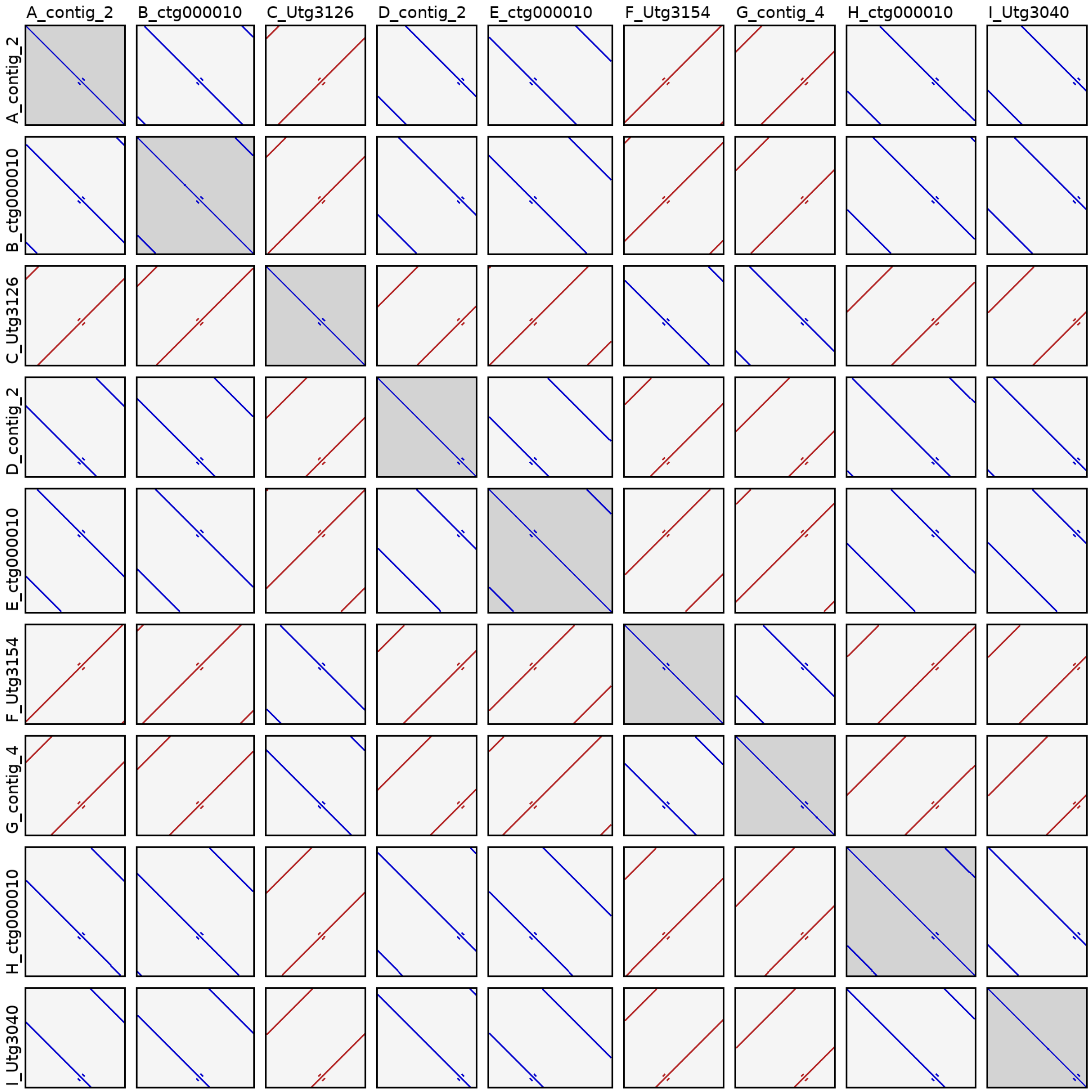

B22FPs1-129 – clustering tree

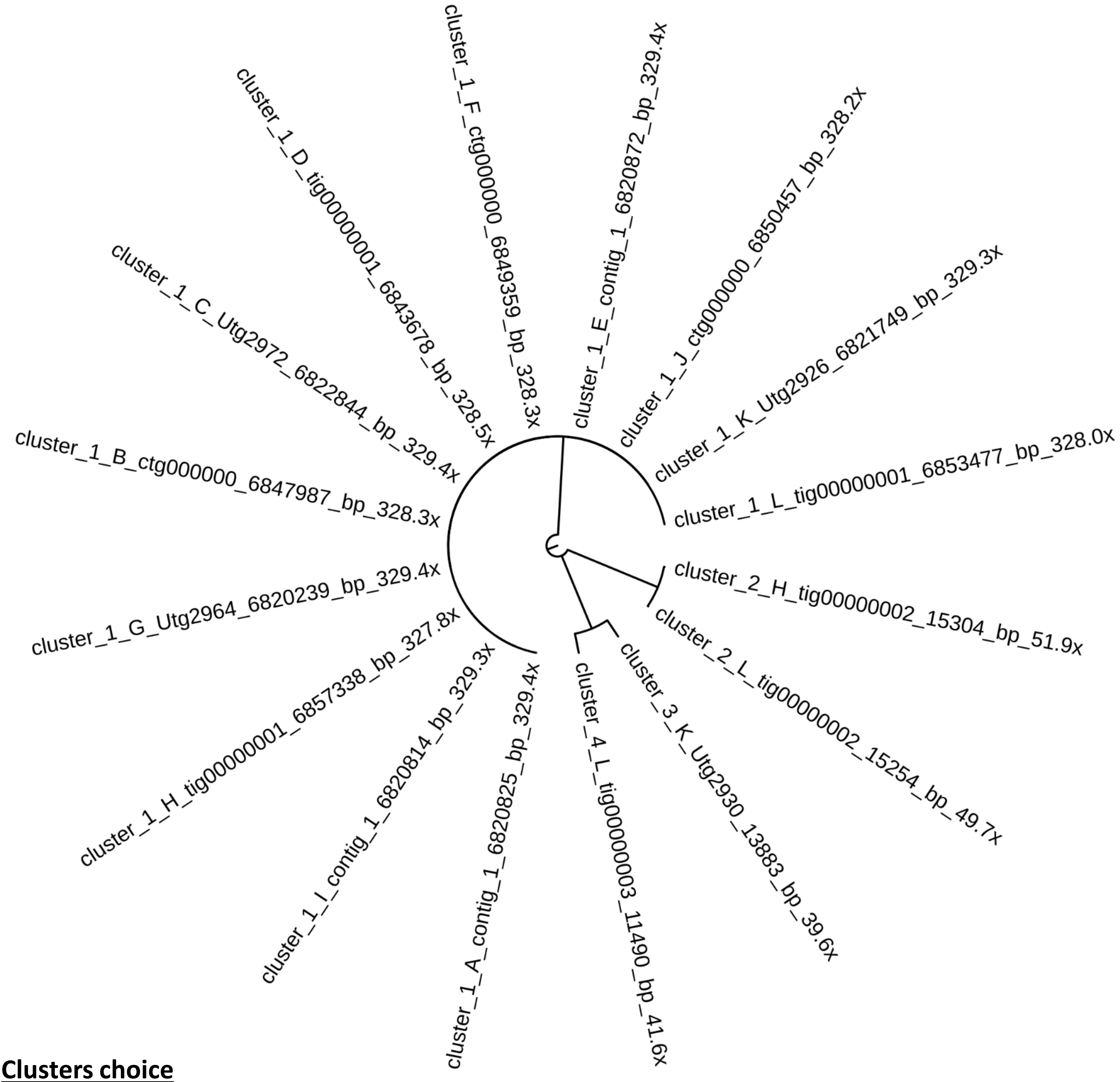

Clusters choice

- **Cluster 1 (chromosome?)**
    - All contigs were chosen
- **Cluster 2, 3 and 4 (plasmids?)**
    - Check for similarities before removing

Reconcile step

❑ **CLUSTER 1 (Chromosome) – 12 contigs**

- **Contig B_ctg000000 was removed due to unability to circularize to other contigs**

  → 11 contigs remaining
- **Contig F_ctg000000 was removed due to unability to circularize to other contigs**

  → 10 contigs remaining
- **Worst-1Kb identity lower than 85%**
    - Contigs C_Utg2972 (38.4%), G_Utg2964 (23.8% - 27.7%) and K_Utg2926 (75.5%) were removed

      → 7 contigs remaining

B26FPs3-140 – clustering tree

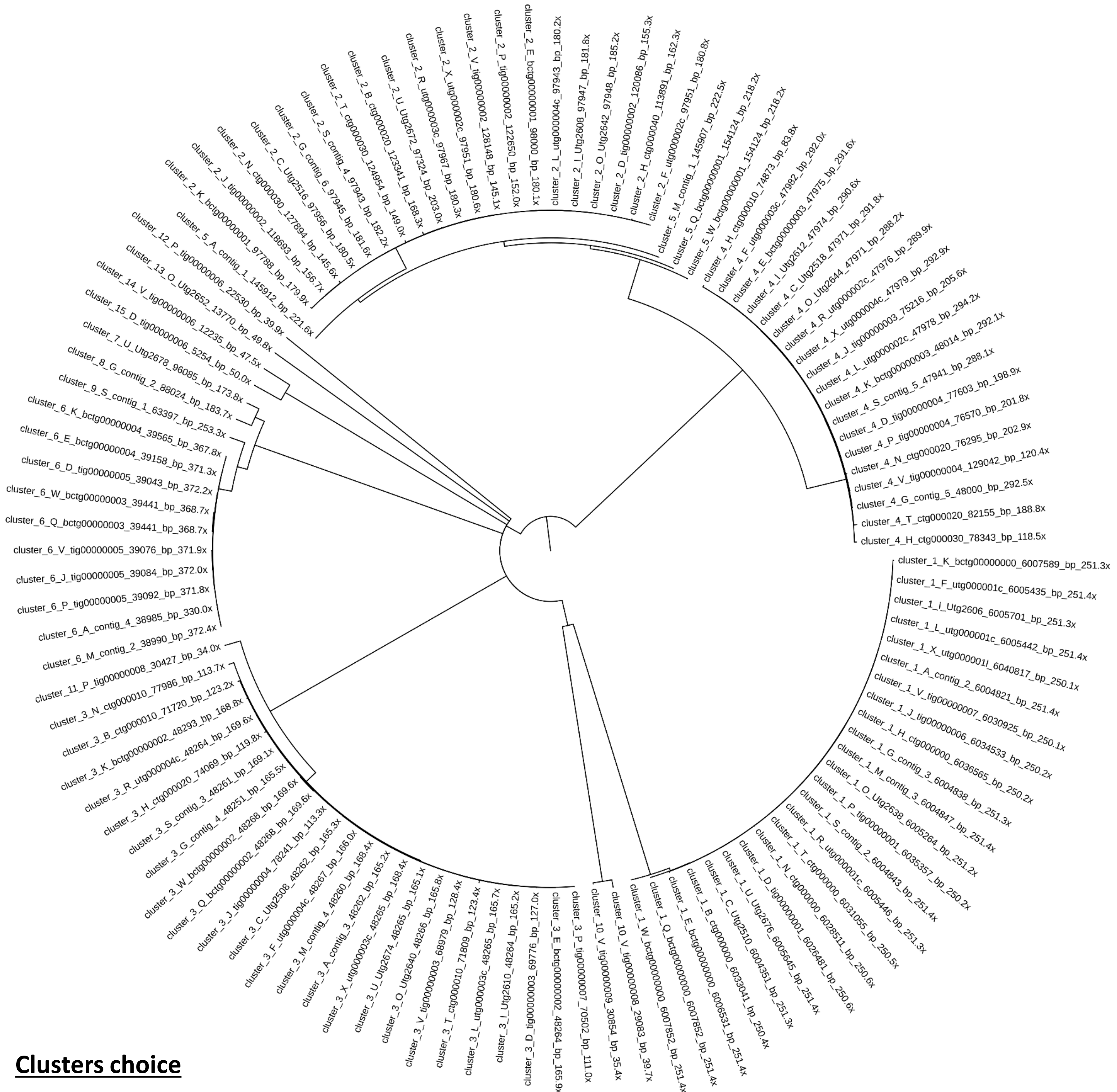

Clusters choice

- **Cluster 1 (chromosome?)**
  - All contigs were chosen
- **Clusters 2, 3 and 4 (plasmids?)**
  - Manual trimming was necessary to be performed
  - Check if manual trimming of cluster 5 can add it in cluster 2?
- **Cluster 6 (plasmid?)**
  - Check if clusters 7, 8 and 9 can be added using manual trimming
- **Clusters 10 and 11**
  - Probably an error from one assembler (only 2 contigs from the same assembler)
- **Clusters 12, 13, 14 and 15**
  - When BLASTed, contigs belonged to phage or plasmid sequences. 3 out of 4 contigs were provided by the same assembler → excluded

B26FPs3-140 – clustering tree

Reconcile step

- CLUSTER 1 (Chromosome) – 24 contigs
  - **Contig B-ctg000000 was removed due to unability to circularize to other contigs**
    → 23 contigs remaining
  - **Contig X_utg000001l was removed due to unability to circularize to other contigs**
    → 22 contigs remaining
  - **Worst-1Kb identity lower than 85%**
    - Contig C_utg2510 (14.4% - 24.7%) was removed
    - Contig E_bctg00000000 (60.6% - 60.7%) was removed
    - Contig I_Utg2606 (68.0%) was removed
    - Contig U_Utg2676 (60.6% - 67.2%) was removed
    → 18 contigs remaining
- CLUSTER 2 (97Kb plasmid) – 20 contigs
  - **All contigs from cluster 5 were excluded due to**
    - Low number (n=4) of contigs found by two assemblers only
    - After manual trimming contig sizes were about 122Kb > 97Kb
  - **Manual trimming**
    - 8 contigs were trimmed (B, D, H, J, N, P, T, V)
  - **Contig D_tig00000002 showed a low overall pairwise identities (< 99%)**
    - Contig removed (95.4% - 95.6%)
    → 19 contigs remaining
  - **Worst-1Kb identity lower than 85%**
    - Contig K_bctg00000001 (81.4%) was removed
    - Contig U_Utg2672 (35.9% - 38.3%) was removed
    → 17 contigs remaining
- CLUSTER 3 (48Kb plasmid) – 24 contigs
  - **Manual trimming**
    - 8 contigs were trimmed (B, D, H, J, N, P, T, V)
- CLUSTER 4 (47Kb plasmid) – 19 contigs
  - **Manual trimming**
    - 8 contigs were trimmed (D, 2xH, J, N, P, T, V)
- CLUSTER 6 (39Kb plasmid) – 10 contigs
  - **All contigs from cluster 7, 8 and 9 were excluded due to**
    - Low number (n=4) of contigs found by two assemblers only
    - After manual trimming contig sizes were about 80Kb > 39Kb
  - **DNA molecule seems linear (see dotplots)**
  - **Contig A_contig_4 was removed due to weak overall pairwise identities (55% - 60%)**
    → 9 contigs remaining
  - **Contigs Q_bctg00000003 (66% - 73%) and W_bctg00000003 (66% - 74.5%) were removed due to worst-1Kb identity (<85%)**
    → 7 contigs remaining
  - **Contigs K_bctg00000004 (54.3% - 66.6%) were removed due to worst-1Kb identity (<85%)**
    → 6 contigs remaining

B26FPs3-140 – clustering tree

- ❑ **CLUSTER 2 (97Kb plasmid) – 20 contigs**

  → **It seems that it is a circular DNA based on dotplots**

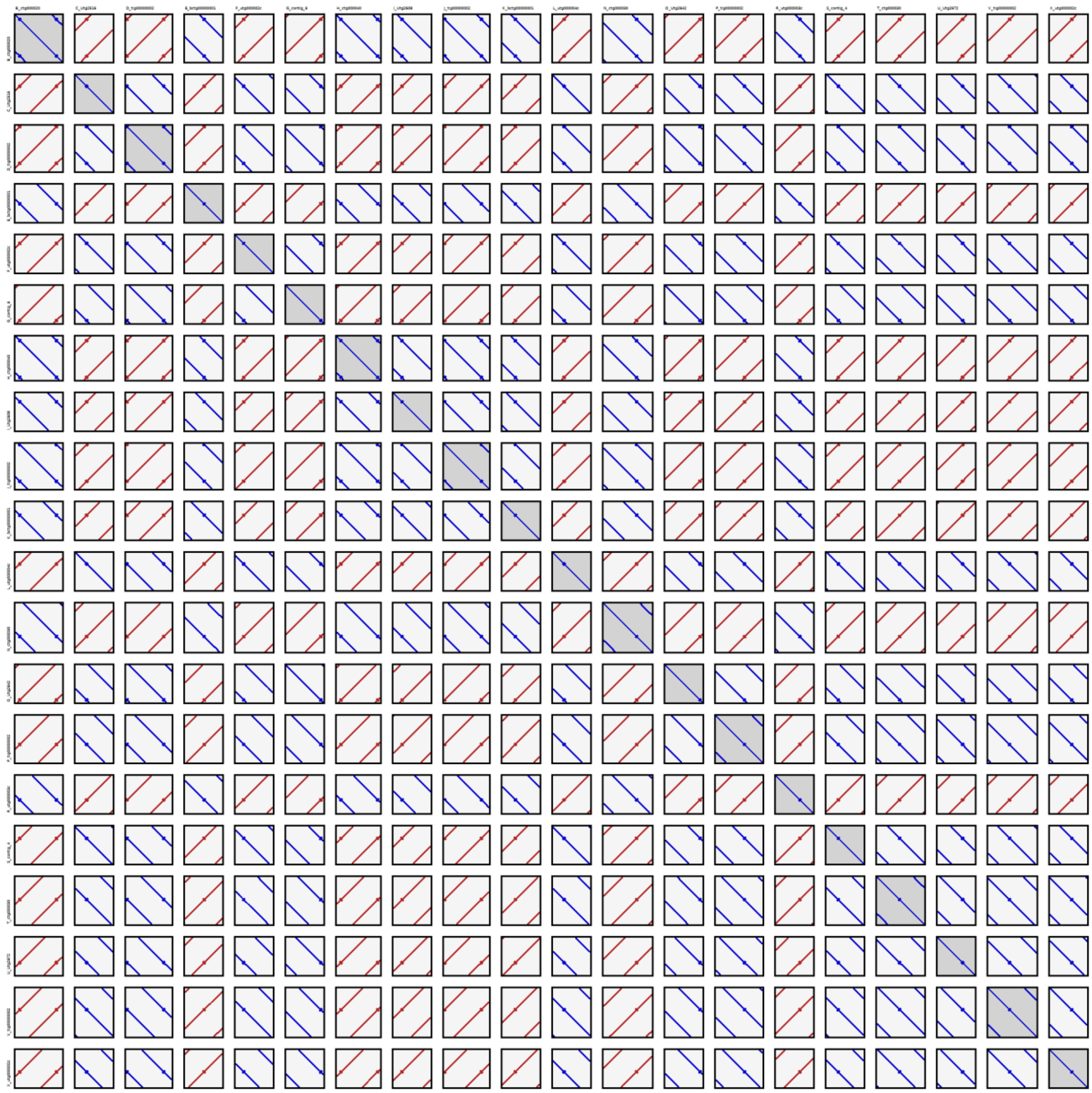

B26FPs3-140 – clustering tree

- ❑ **CLUSTER 3 (48Kb plasmid) – 24 contigs**

→ It seems that it is a circular DNA based on dotplots

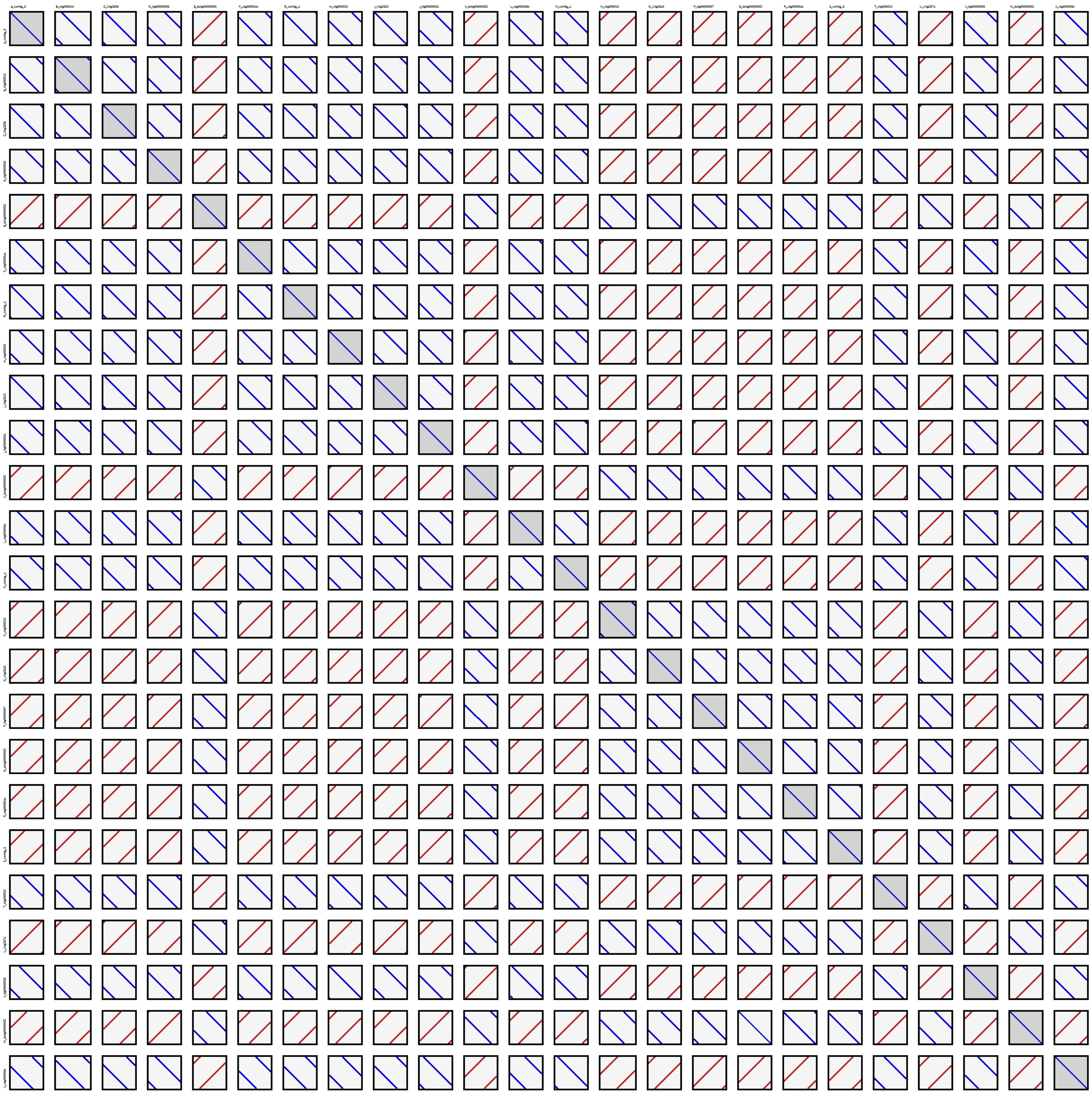

B26FPs3-140 – clustering tree

❑ **CLUSTER 4 (47Kb plasmid) – 19 contigs**

→ It seems that it is a circular DNA based on dotplots

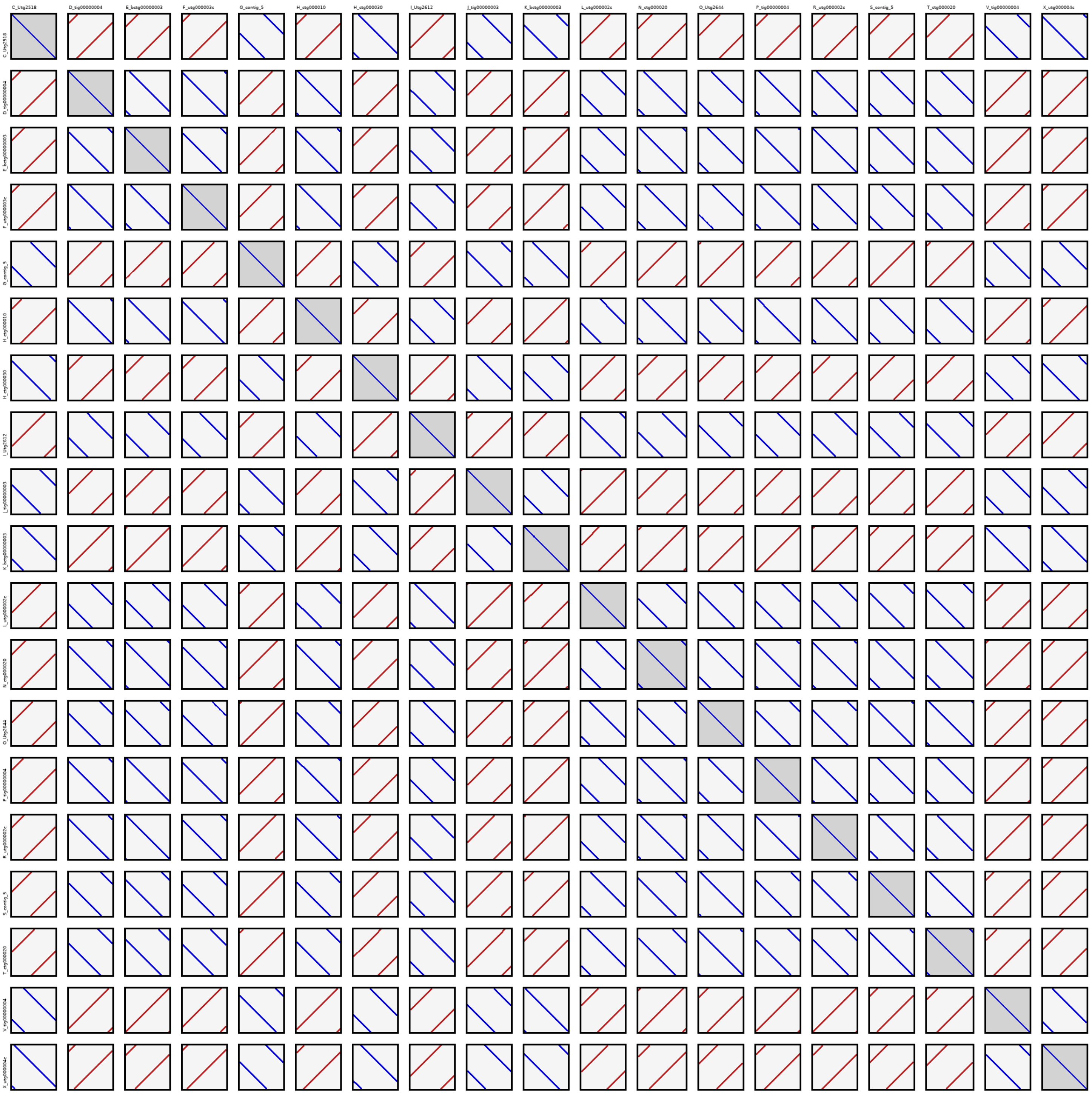

B26FPs3-140 – clustering tree

- ❑ **CLUSTER 6 (47Kb plasmid) – 19 contigs**

  → It seems that it is a linear DNA based on dotplots

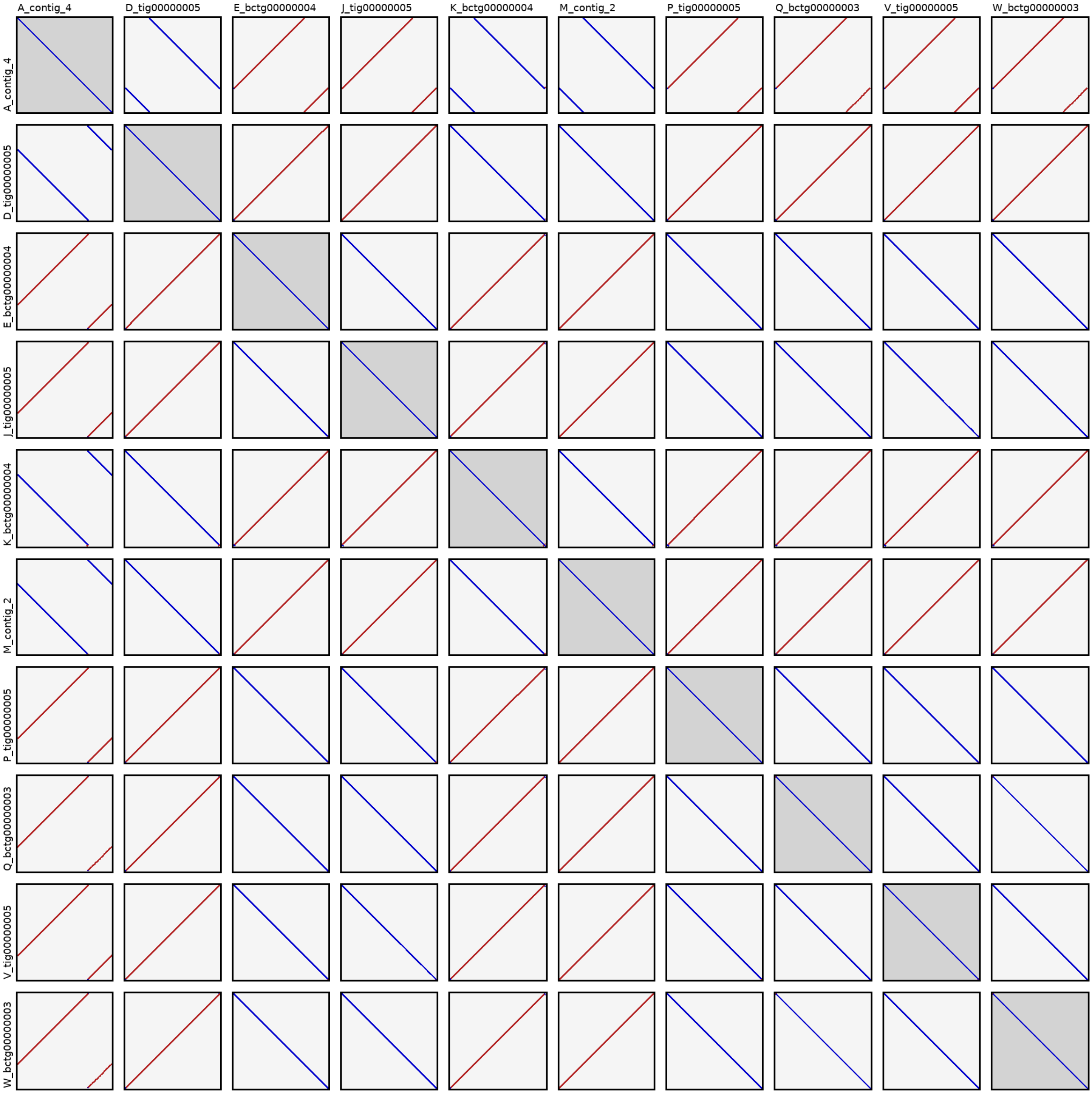

Supplement: Uncited Supplementary Material 1. [file acmi-8-01029-s001.pdf]
